# Cranial Anatomy and Palaeoneurology of the Archosaur *Riojasuchus tenuisceps* from the Los Colorados Formation, La Rioja, Argentina

**DOI:** 10.1371/journal.pone.0148575

**Published:** 2016-02-05

**Authors:** Maria Belen von Baczko, Julia Brenda Desojo

**Affiliations:** 1 Consejo Nacional de Investigaciones Científicas y Técnicas (CONICET), Ciudad Autónoma de Buenos Aires, Buenos Aires, Argentina; 2 Sección Paleontología Vertebrados, Museo Argentino de Ciencias Naturales, Ciudad Autónoma de Buenos Aires, Buenos Aires, Argentina; Raymond M. Alf Museum of Paleontology, UNITED STATES

## Abstract

*Riojasuchus tenuisceps* Bonaparte 1967 is currently known from four specimens, including two complete skulls, collected in the late 1960s from the upper levels of the Los Colorados Formation (Late Triassic), La Rioja, Argentina. Computed tomography (CT) scans of the skulls of the holotype and a referred specimen of *Riojasuchus tenuisceps* and the repreparation of the latter allows recognition of new features for a detailed analysis of its cranial anatomy and its comparison with a wide variety of other archosauriform taxa. The diagnosis of *Riojasuchus tenuisceps* is emended and two autapomorphies are identified on the skull: (1) a deep antorbital fossa with its anterior and ventral edges almost coinciding with the same edges of the maxilla itself and (2) a suborbital fenestra equal in size to the palatine-pterygoid fenestra. Also, the first digital 3D reconstruction of the encephalon of *Riojasuchus tenuisceps* was carried out to study its neuroanatomy, showing a shape and cranial nerve disposition consistent to that of other pseudosuchians.

## Introduction

Ornithosuchidae is a clade of pseudosuchian archosaurs known from Upper Triassic continental beds along with aetosaurs, gracilisuchids, “rauisuchians”, and basal crocodylomorphs [1, 2]. Ornithosuchids are terrestrial quadrupedal carnivorous archosaurs with sizes ranging from 2 to 4 m. They have distinctive features such as a strongly downturned premaxilla, a two-tooth diastema between the premaxilla and maxilla, the lower jaws shorter than the skull, the presence of a palatine-pterygoid fenestra, and a unique “crocodile-reversed” ankle articulation, only known in this group, which consists of a tarsal articulation with a ventral concavity on the astragalus and a convexity on the calcaneum [3,4,5]. Ornithosuchidae currently comprises three species: *Riojasuchus tenuisceps* [6], *Venaticosuchus rusconii* [7], and *Ornithosuchus longidens* [8]. The first two are known from the Ischigualasto-Villa Union Basin, La Rioja, Argentina, and the latter from the Lossiemouth Sandstones Formation (late Carnian–early Norian [9]), Moray, Scotland.

*Riojasuchus tenuisceps* is presently represented by four individuals of similar size found in the Los Colorados Formation (Norian [10]), La Rioja, Argentina. They were collected from the upper section of this formation and are part of the very rich and diverse Coloradian fauna [11]. The species was erected by Bonaparte in 1967 and later described by the same author in 1972, highlighting the similarities and differences with its Scottish relative *Ornithosuchus longidens*. The latter is the only ornithosuchid known from the northern hemisphere (Lossiemouth Sandstones Formation, Moray, Scotland) and consists of 11 specimens from different ontogenetic stages, preserved mainly as moulds along with some cranial and skeletal three-dimensional elements. It was the first ornithosuchid to be described [8], later on utilized by Huene [12] to erect the clade Ornithosuchidae, and reassessed and redescribed in great detail by Walker [13]. In 1970, *Venaticosuchus rusconii* was briefly described by Bonaparte as a new ornithosuchid because of its overall similarities with *Riojasuchus tenuisceps* and *Ornithosuchus longidens*. *Venaticosuchus rusconii* was collected from the Ischigualasto Formation (late Carnian–early Norian [14]), La Rioja, Argentina, and is represented only by a fragmentary skull. It was recently described in detail and included for the first time within a quantitative analysis to test its phylogenetic affinities by Baczko 2012 [15] and Baczko et al. 2014 [4].

For this contribution, a detailed anatomical study of the skull of *Riojasuchus tenuisceps* is carried out revealing some of its distinctive features. New information is detailed about its previously unknown neuroanatomy, provided by the CT scans that we performed on both preserved skulls (PVL 3827, 3828). This contribution is one result of a broader project on the anatomy of Ornithosuchidae, which has already included an overview on the general aspects of the anatomy and evolution of this clade [3], a detailed redescription of *Venaticosuchus rusconii* [4], and a preliminary study of the olfactory cavities of *Riojasuchus tenuisceps* and *Venaticosuchus rusconii* [16]. We hope it will enrich upcoming studies on the skeletal anatomy and phylogenetic relationships of Ornithosuchidae.

## Horizon and Locality

**Fig 1:**
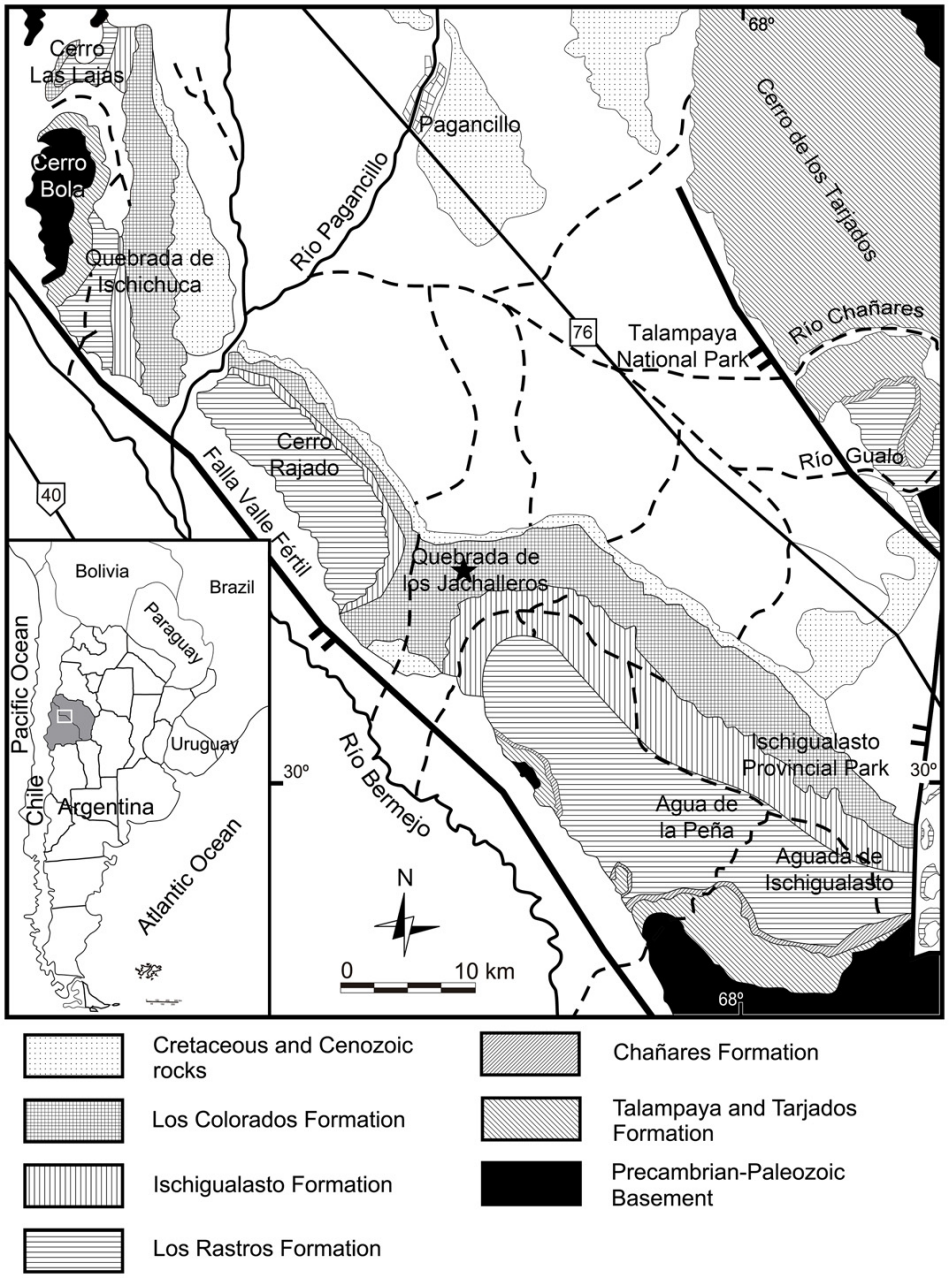
Geological map of the Los Colorados Formation, Ischigualasto-Villa Unión Basin, La Rioja, Argentina. Star indicates the location where the specimens were collected. Modified from Baczko et al. 2014.

The fossils described here were collected in the upper beds of the Los Colorados Formation (Late Triassic), Quebrada de los Jachalleros (= Jachaleros; [17,18]), General Lavalle, La Rioja province, NW Argentina (Fig 1). This upper section of the Los Colorados Formation has yielded one of the most diverse and abundant faunas of the Late Triassic, the basis for the Coloradian (“Coloradense”) fauna [11]. The Coloradian fauna is mainly composed of pseudosuchians (ornithosuchids, “rauisuchians”, sphenosuchians, and protosuchians), avemetatarsalians (sauropodomorph and theropods), quelonids, and cynodonts. The available paleomagnetochronologic studies date the Los Colorados Formation at 227–213 Ma, corresponding to a Norian age [10].

## Materials and Methods

*Riojasuchus tenuisceps* is currently represented by four specimens, two of which have almost completely preserved skulls (PVL 3827, 3828). The skull of PVL 3827 was scanned at the Clínica La Sagrada Familia, Buenos Aires, on a 64-channel axial CT scaner and the skull of PVL 3828 was scanned at MATSA, San Miguel de Tucumán, on a 16-channel axial CT scaner. In both cases, the settings were: field of view 421.0 mm, penetration power of 120.0 Kv and 279 mA, slice thickness of 0.8 mm and 0.4 mm of overlap. For the analysis of the CT images and 3D reconstruction, we used the open source software 3D Slicer v4.1.1. Terminology used for the description of the digital endocast does not refer strictly to the soft tissue regions of the brain because, as seen in modern archosaurs (e.g. *Alligator mississippiensis*: OUVC 9761), the endocast also includes the volume occupied by other tissues surrounding the brain (e.i. vascular tissue) (e.g. [19, 20])

The skull of PVL 3828 was loaned by the Instituto Miguel Lillo, Tucumán, with the agreement of the curator Jaime Powell for its mechanical preparation with a micro jackhammer and its study at the Museo Argentino de Ciencias Naturales “Bernardino Rivadavia”, Buenos Aires. No specimens were purchased or donated for the purpose of this study. Measurements were made with a digital caliper set with a maximum deviation of 0.02 mm but measurements were rounded to the nearest 0.1 millimeter. Lengths were measured according to the anteroposterior axis of the elements of the skull, heights following the dorsoventral axis perpendicular to the lengths, and widths were measured following the mediolateral axis of the skull. Skull length was measured from the premaxilla to the quadrate.

All specimens studied first-hand for comparative purposes (indicated by the citation of their taxonomic name and respective collection accession numbers) were studied with the permission of appropriate curators and/or collection managers (see Acknowledgements), in recognized, scientifically accessible collections. Repository locations and abbreviations for all specimens discussed in the text are as follows:

**BSPG**, Bayerische Staatssammlung für Paläontologie und Geologie, Münich, Germany; **CPEZ**, Coleção Municipal, São Pedro do Sul, Brazil; **EM**, Elgin Museum, Elgin, Scotland; **GPIT**, Institut und Museum für Geologie und Paläeontologie der Universität Tübingen, Tübingen, Germany; **MACN-HE**, División Herpetología, Museo Argentino de Ciencias Naturales Bernardino Rivadavia, Buenos Aires, Argentina; **MCZD**, Marischal College, Zoology Department, Aberdeen, Scotland; **NHMUK PV R**, Natural History Museum, London, UK; **OUVC**, Ohio University, Vertebrate Collection, Athens, Ohio, USA: **PULR**, Museo de Paleontología, Universidad Nacional de La Rioja, La Rioja, Argentina; **PVL**, Paleontología de Vertebrados, Instituto Miguel Lillo, Tucumán, Argentina; **PVSJ**, Paleontología, Museo de Ciencias Naturales, Universidad Nacional de San Juan, San Juan, Argentina; **SAM-PK**, Iziko South African Museum, Cape Town, South Africa; **SMNS**, Staatliches Museum für Naturkunde, Stuttgart, Germany; **TMM**, Texas Memorial Museum, Austin, Texas, USA; **UFRGS-PV**, Departamento de Paleontologia e Estratigrafia, Instituto de Geociências, Universidade Federal do Rio Grande do Sul, Brazil; **ZPAL**, Institute of Paleobiology, Polish Academy of Sciences, Warsaw.

### Systematic Palaeontology

Archosauria Cope 1869 [21] sensu Gauthier and Padian 1985 [22]

Pseudosuchia Zittel 1887–1890 [23] sensu Gauthier and Padian 1985 [22]

Ornithosuchidae Huene 1908 [12] sensu Sereno 1991 [5]

*Riojasuchus* Bonaparte 1967 [6]

*Riojasuchus tenuisceps* Bonaparte 1967 [6]

Figs 2–7

**Fig 2:**
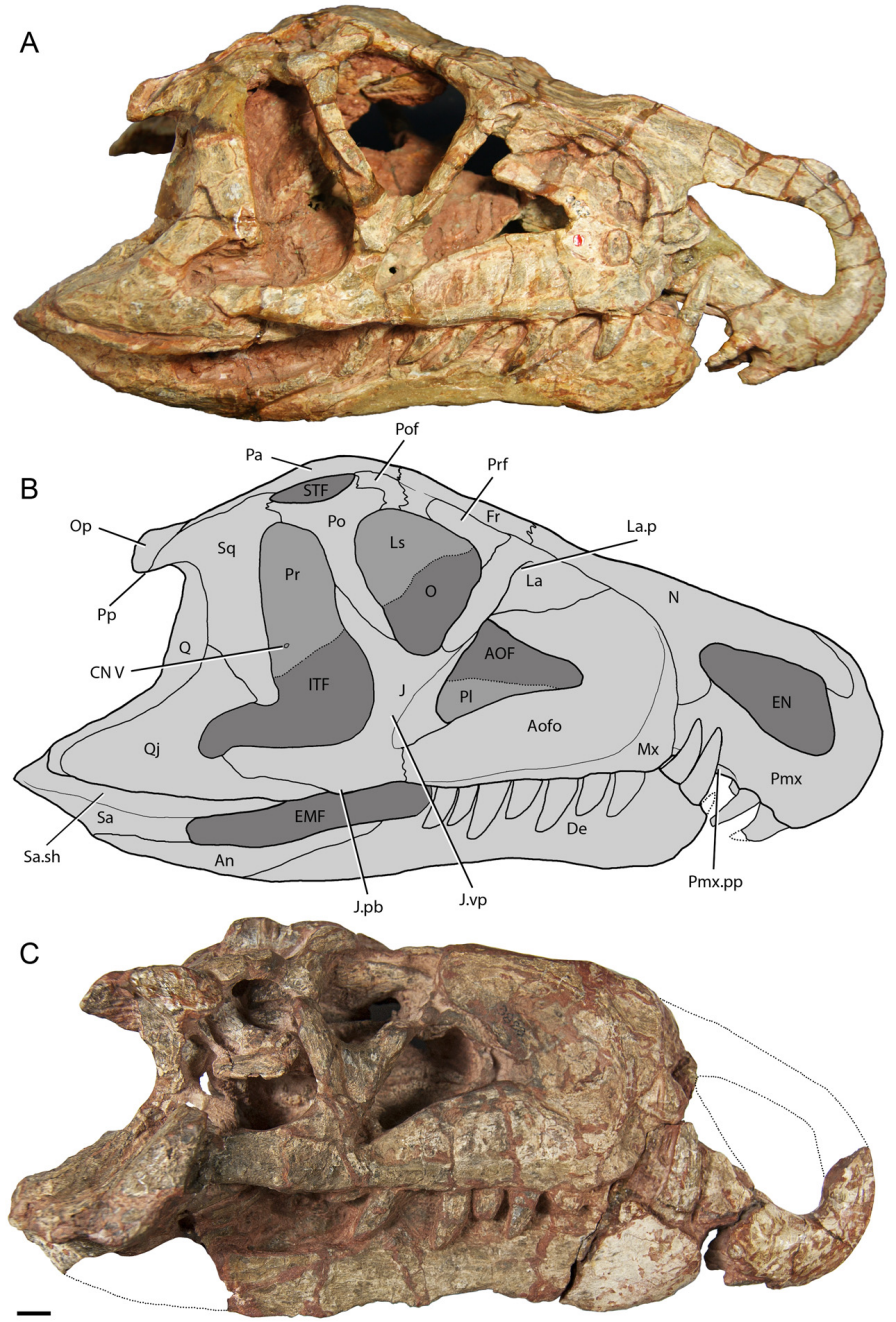
Skulls of *Riojasuchus tenuisceps* in right lateral view. (A) Holotype PVL 3827; (B) reconstruction based on both specimens; (C) Referred specimen PVL 3828. *Abbreviations*: An, angular; AOF, antorbital fenestra; Aofo, antorbital fossa; CN V, exit for cranial nerve V; De, dentary; EMF, external mandibular fenestra; EN, external nares; Fr, frontal; ITF, infratemporal fenestra; J, jugal; J.pb, protuberance of the jugal; J.vp, vertical process of the jugal; La, lacrimal; La.p, pocket on the lacrimal; Ls, laterosphenoid; Mx, maxilla; N, nasal; O, orbit; Op, opisthotic; Pa, parietal; Pfr, prefrontal; Pl, palatine; Pmx, premaxilla; Pmx,pp, palatal process of the premaxilla; Po, postorbital; Pof, postfrontal; Pp, paroccipital process; Pr, prootic; Q, quadrate; Qj, quadratojugal; Sa, surangular; Sa.sh, surangular shelf; Sq, squamosal; STF, supratemporal fenestra. Scale bar: 1cm.

**Fig 3:**
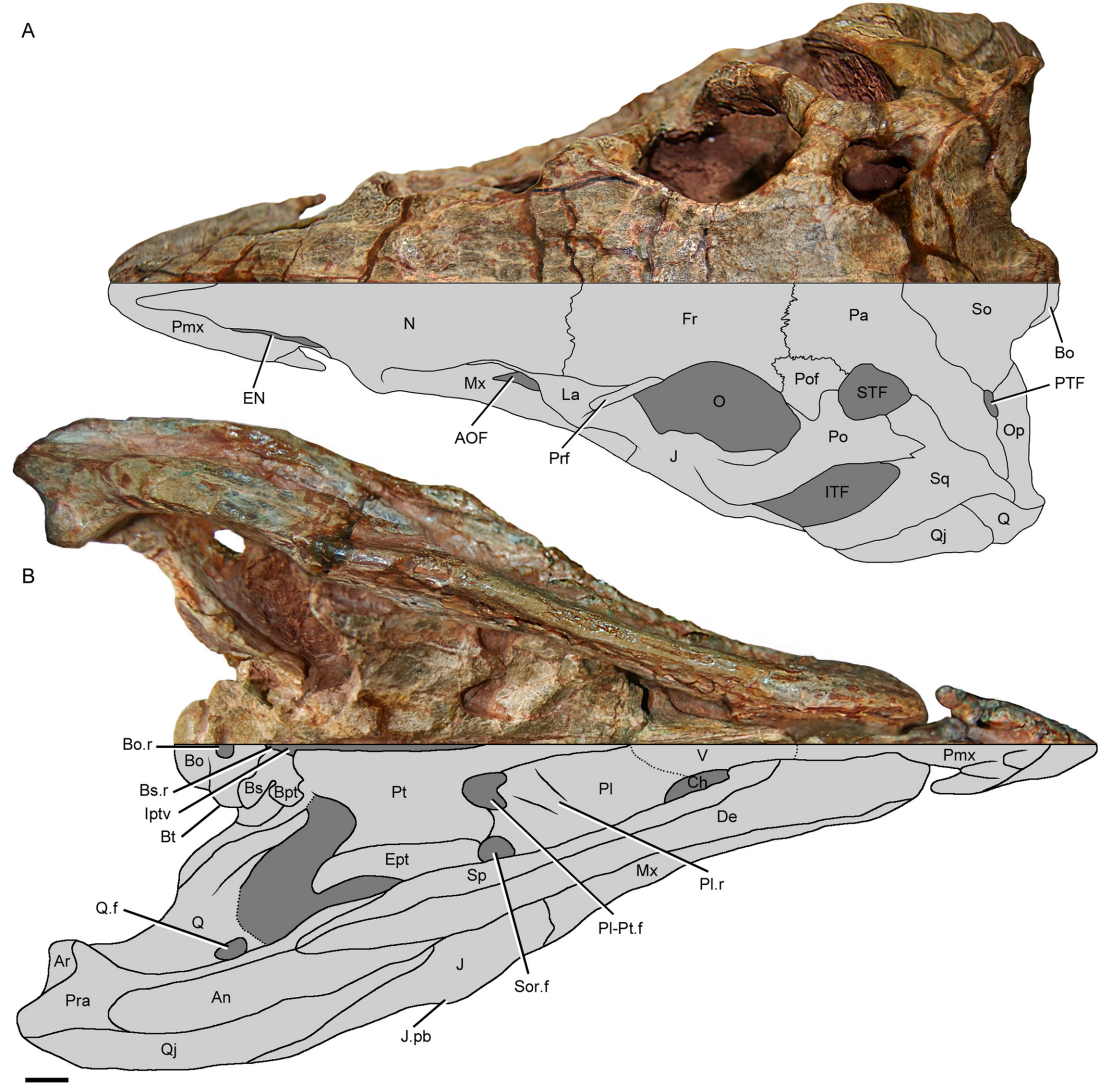
Holotype skull of *Riojasuchus tenuisceps* in dorsal and ventral view. (A) dorsal view of PVL 3827; (B) ventral view of PVL 3827; (C) detail of possible palatine teeth. Lower halves as interpretative drawing. *Abbreviations*: AOF, antorbital fenestra; An, angular; Ar, articular; Bo, basioccipital; Bo.r, basioccipital recess; Bpt, basipterigoid process; Bs, basisphenoid; Bs.r, basisphenoid recess; Bt, basal tubera; Ch, choana; De, dentary; EN, external nares; Ept, ectopterygoid; Fr, frontal; Iptv, interpterygoid vacuity; ITF, infratemporal fenestra; J, jugal; J.pb, protuberance of the jugal; La, lacrimal; Mx, maxilla; N, nasal; O, orbit; Op, ophistotic; Pa, parietal; Pfr, prefrontal; Pl, palatine; Pl-Pt-f, palatine-pterygoid fenestra; Pl.r, palatine ridge; Pmx, premaxilla; Po, postorbital; Pof, postfrontal; Pra, prearticular; Pt, pterygoid; Q, quadrate; Q.f, quadrate foramen; Qj, quadratojugal; Sp, splenial; Sq, squamosal; STF, supratemporal fenestra; So, supraoccipital; Sor.f, Suborbital fenestra; V, vomer. Scale bar: 1cm.

**Fig 4:**
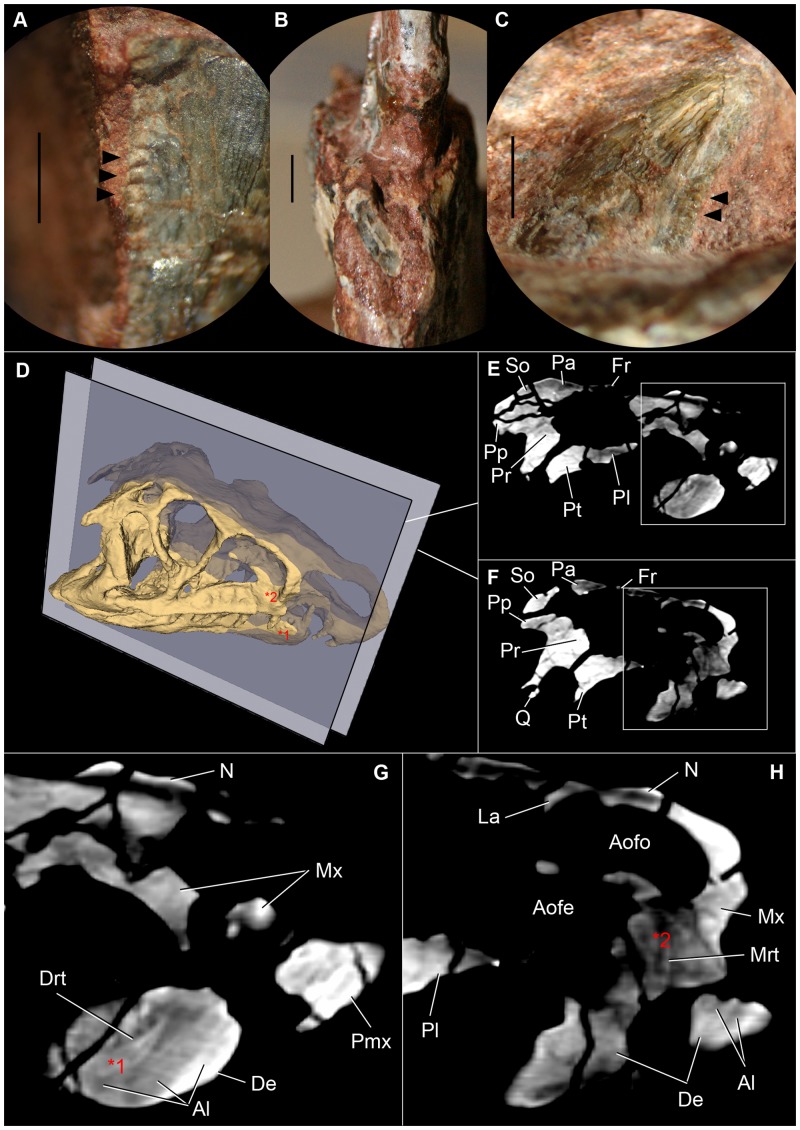
Details of dentition of *Riojasuchus tenuisceps*. (A) lateral detail of denticles on distal margin of maxillary tooth (PVL 3828); (B) anterior view of first dentary tooth (PVL 3827) broken at its base; (C) medial view of posterior dentary tooth (PVL 3828) with denticles on distal margin; (D) 3D reconstruction of the skull of PVL 3827 indicating selected slices; (E-F) selected slices of PVL 3827; (G) detail of slide E indicating a maxillary replacement tooth (Mrt) erupting; (H) detail of slide F pointing out a dentary replancement tooth (Drt); *1 and *2 indicate the position of each replacement tooth on the skull. Arrowheads point at denticles. *Abbreviations*: Al, alveoli; Aofe, antorbital fenestra; Aofo, antorbital fossa; De, dentary, Drt, dentary replacement tooth; Fr, frontal; N, nasal; Mrt, maxillary replacement tooth; Mx, maxilla; Pa, parietal; Pl, palatine; Pmx, premaxilla; Pp, paroccipital process; Pr, prootic; Pt, pterygoid; Q, quadrate; So, supraoccipital. Scale bar: 2mm.

**Fig 5:**
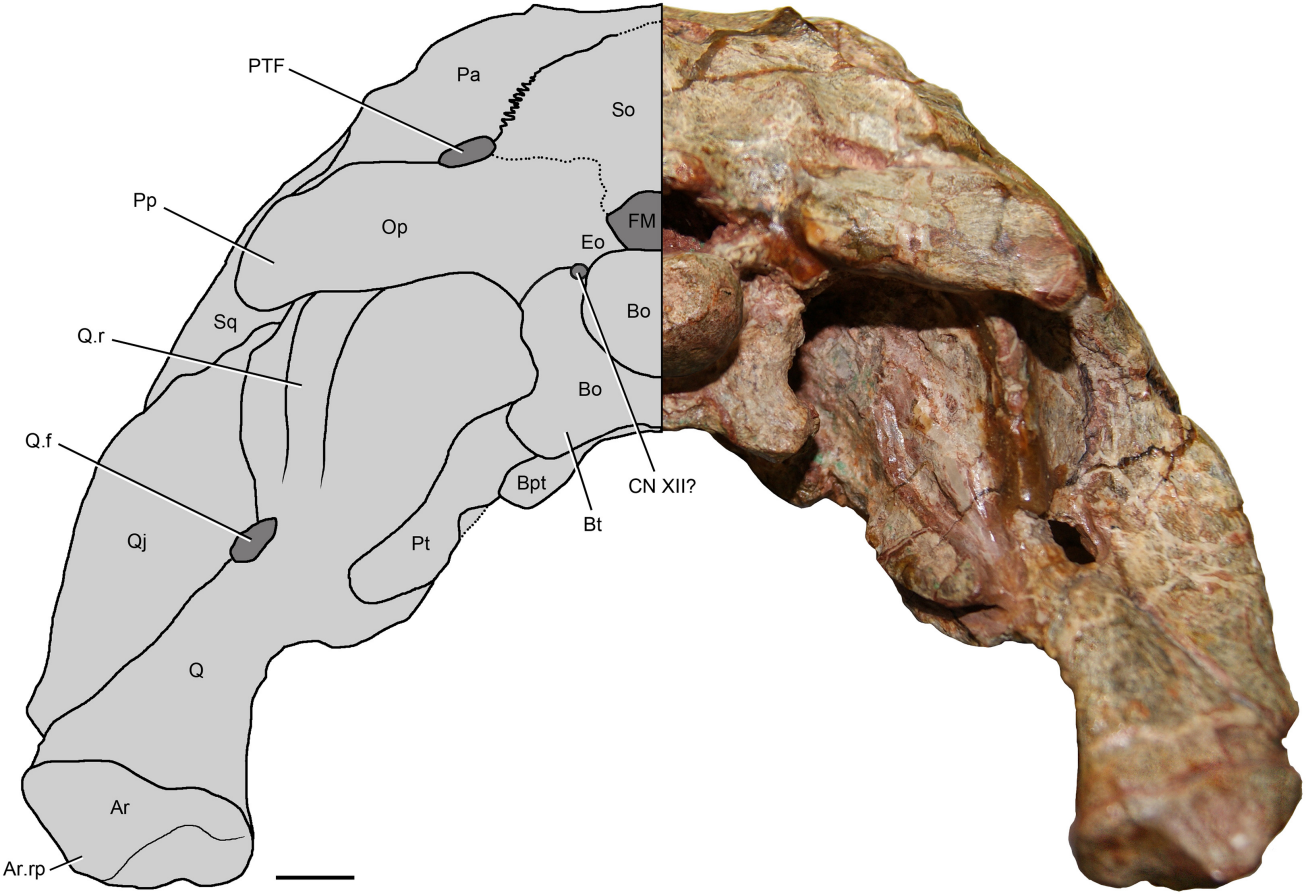
Holotype skull of *Riojasuchus tenuisceps* PVL 3827 in occipital view. Left half as interpretative drawing. *Abbreviations*: Ar, articular; Ar,rp, retroarticular process of the articular; Bo, basioccipital; Bpt, basipterygoid process; Bt, basal tubera; CN XII?, possible exit for cranial nerve XII; Eo, exoccipital; FM, foramen magnum; Op, opisthotic; Pa, parietal; Pp, paroccipital process; Pt, pterygoid; PTF, posttemporal fenestra; Q, quadrate; Q.f, quadrate foramen; Q.r, quadrate ridge; Qj, quadratojugal; Sq, squamosal; So, supraoccipital. Scale bar: 1cm.

**Fig 6:**
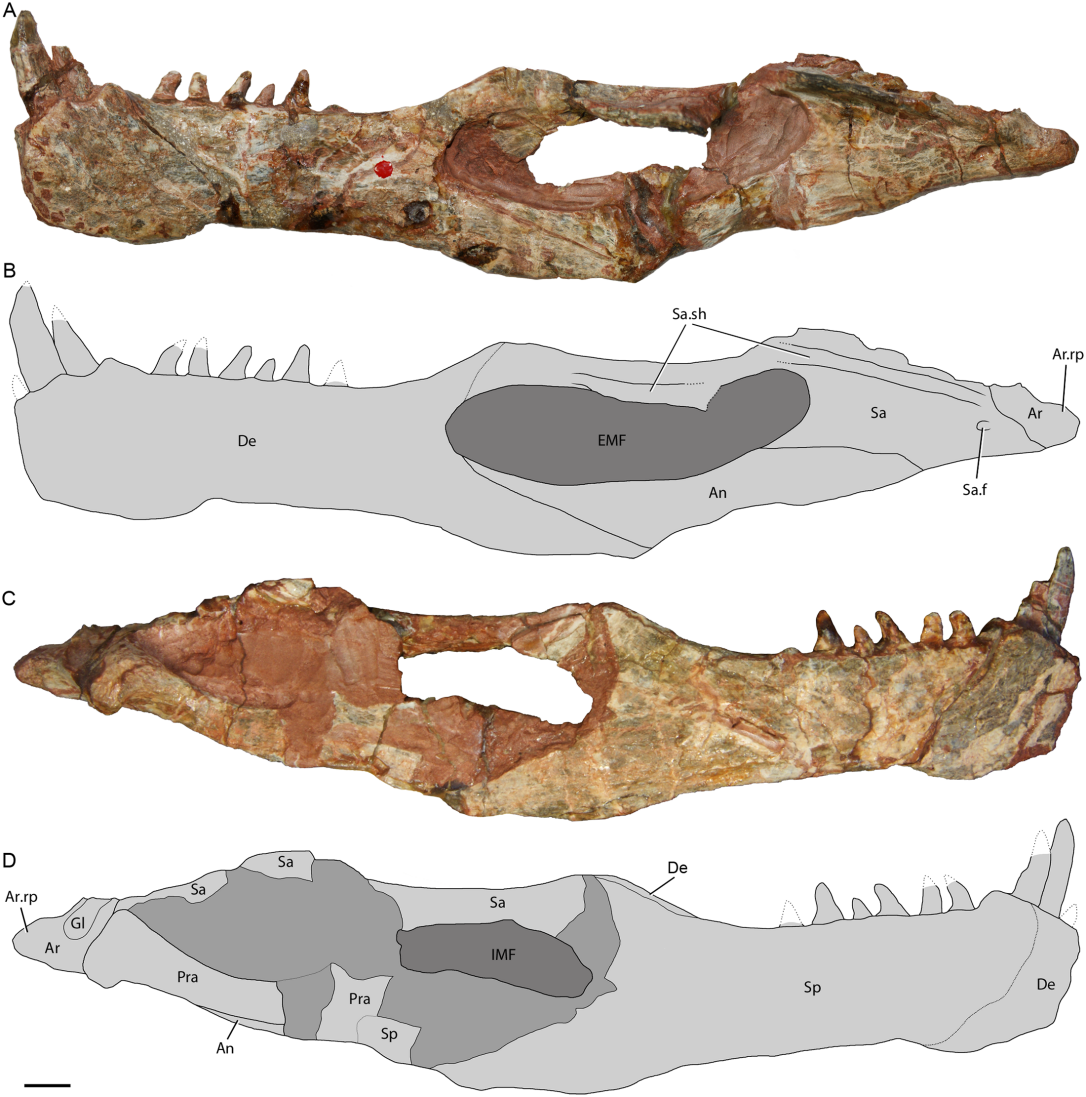
Left hemimandible of holotype of *Riojasuchus tenuisceps* PVL 3827. (A) lateral view; (B) medial view. With interpretative drawings. *Abbreviations*: An, angular; Ar, articular; Ar.rp, retroarticular process of the articular; De, dentary; EMF, external mandibular fenestra; Gl, glenoid fossa; IMF, internal mandibular fenestra; Pra, prearticular; Sa, surangular; Sa.f, surangular foramen; Sa.sh, surangular shelf; Sp, splenial. Scale bar: 1cm.

**Fig 7:**
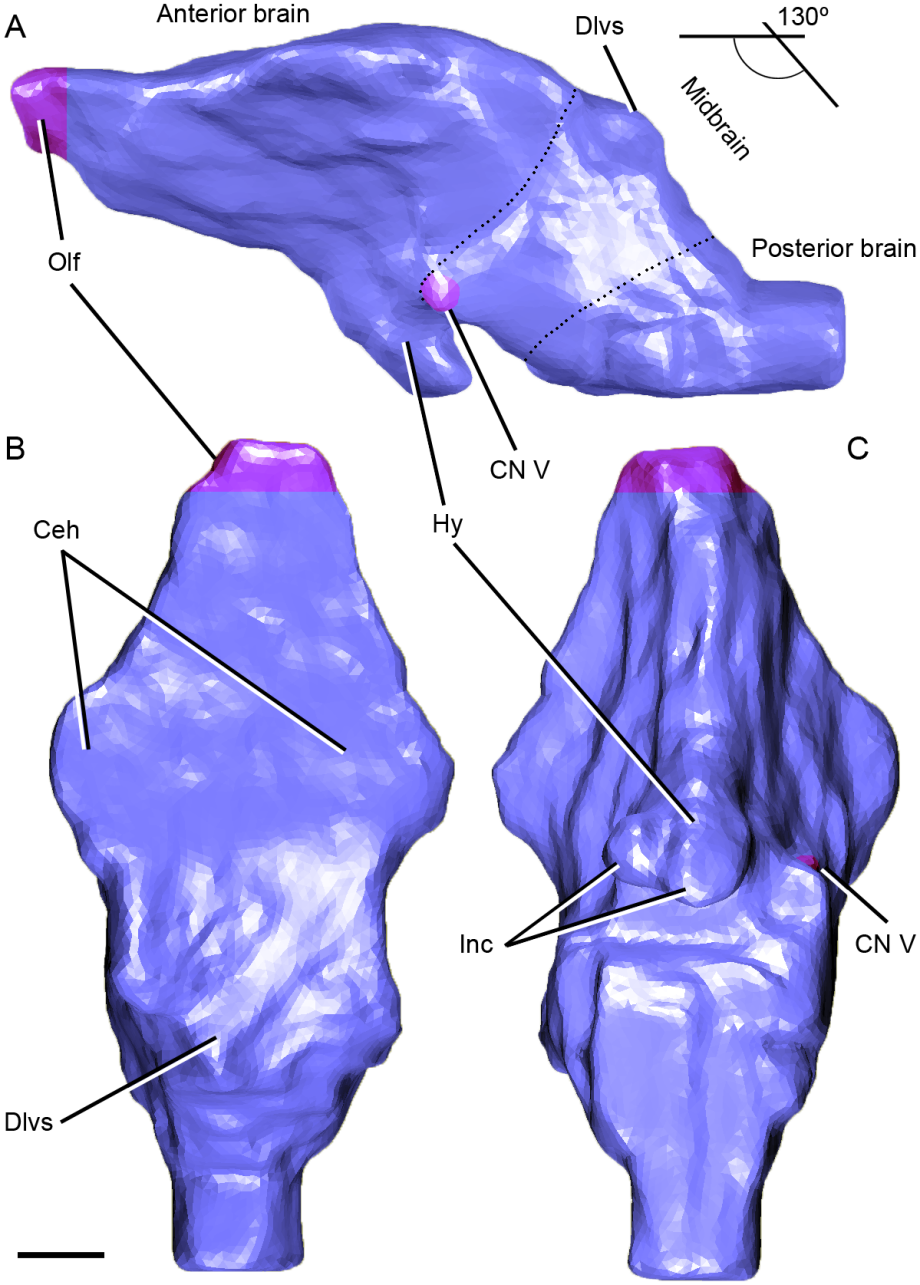
Digital endocast of the braincase of *Riojasuchus tenuisceps* PVL 3827. (A) lateral, (B) dorsal, and (C) ventral view. *Abbreviations*: Ceh, cerebral hemispheres; CN V, cranial nerve V (trigeminal); Dlvs, dorsal longitudinal venous sinus; Hy, hypophysis; Inc, internal carotid arteries; Olf, olfactory tracts. Scale bar: 1cm.

#### Holotype

PVL 3827: The specimen has a complete and very well preserved skull and postcranial elements. The skull has the complete lower jaws, with the right hemimandible articulated and the left hemimandible disarticulated. The skeleton comprises 26 partially articulated vertebrae (19 presacral, 3 sacral, and 4 caudal), with articulated paramedial osteoderms in some cervical and dorsal regions; incomplete scapulocoracoids, incomplete humeri, a distal portion of left radius and ulna articulating with carpus; left ilium and pubis, left femur, left tibiae and fibulae articulating with complete pes.

#### Referred material

PVL 3828: This referred material is a skull with both articulated lower jaws that lacks part of the premaxillae and nasals, left jugal and lachrymal, both squamosals, left surangular, and right angular and articular. Moreover, this specimen includes several skeletal elements including 32 partially articulated vertebrae (20 presacral, 12 caudal); incomplete scapulocoracoids, humeri, radius, and ulnae; one carpal element isolated; left ilium and a fragmentary ischium; left femur; incomplete right femur, tibiae and fibulae; left calcaneum and several disarticulated phalanxes.

PVL 3826: This specimen is represented only by postcranial elements, namely 28 vertebrae (19 presacral and 9 caudal) partially articulated, two fragmentary scapulocoracoids, right humerus, fragments of the radius and ulna, incomplete right ilium, distal end of the left femur, and a fragmentary tibia.

PVL 3814: This specimen consists of postcranial material, such as some poorly preserved isolated vertebrae, proximal end of a humerus, fragment of a tibia, and several isolated osteoderms.

#### Emended Diagnosis

Ornithosuchid archosaur distinguished from other ornithosuchids by the following autapomorphies: (1) deep antorbital fossa with the anterior and ventral edges almost coinciding with the same edges of the maxilla itself; (2) suborbital fenestra equal in size to the palatine-pterygoid fenestra; (3) atlantal neural arch bases contact at the midline. *Riojasuchus tenuisceps* is distinguished from all other archosauriforms by the combination of the following features: (1) strongly down-turned premaxilla; (2) three premaxillary teeth; (3) 7 maxillary teeth; (4) second and third teeth on dentary hypertrophied; (5) two-tooth diastema between premaxilla and maxilla; (6) nasal-prefrontal contact absent; (7) jugal with vertical process separating antorbital fenestra from infratemporal fenestra; (8) orbit with ventral point surrounded by V-shaped dorsal processes of the jugal; (9) posterolateral process of the parietals anteriorly inclined greater than 45°; (10) reduced supratemporal fenestra; (11) L-shaped infratemporal fenestra; (12) presence of a palatine-pterygoid fenestra; (13) lower jaws shorter than skull; (14) presence of a first small tooth anterior to the two hypertrophied teeth; (15) anterior end of the dentary dorsally expanded; (16) dentary-splenial symphysis present along one-third of the lower jaw; (17) sharp surangular shelf; (18) presence of a surangular foramen; (19) ventral keel of cervical vertebrae extends ventral to the centrum rims; (2) pubis longer than 70% of femoral length; (21) anterior trochanter (= M. iliofemoralis cranialis insertion) forms a steep margin with the shaft but is completely connected to the shaft; (22) ventral astragalocalcanear articular surface concavoconvex with concavity on astragalus; (23) metatarsal V without ‘‘hooked” proximal end.

## Results

### Description and Comparisons

*Riojasuchus tenuisceps* is represented by four specimens, but only two of them have associated skulls (PVL 3827, 3828), and on which we base our descrption. The skull of the holotype suffered some damage during its original mechanical preparation, probably because of the technology available during the 1970s when it was unearthed. The second skull (PVL 3828) has the posterior region of the skull roof crushed, but it was less damaged during its original preparation. This material was reprepared at the Museo Argentino de Ciencias Naturales “Bernardino Rivadavia” during 2012, which allow us to observe in detail new data for some of the regions that are damaged in the holotype specimen.

For this study, comparisons were made with original material of fossil archosauriforms studied first hand by both authors and through the primary literature, both listed in Table 1.

**Table 1:** List of comparative material.

Taxon	Reference	Reference Number
*Proterosuchus fergusi*	Gow 1975[24]; Welman 1998[25]; Ezcurra and Butler 2015[26]	-
*Erythrosuchus africanus*	Gower 1997[27], 2003[28]	-
*Garjainia madiba*	Gower et al. 2014[29]	-
*Doswellia kaltenbachi*	Weems 1980[30]; Dilkes and Sues 2009[31]	-
*Chanaresuchus bonapartei*	Romer 1971[32]; Trotteyn and Haro 2012[33]	PULR 07; PVL 4575, 4586, 4647
*Gualosuchus reigi*	Romer 1971[32]; Dilkes and Arcucci 2012[34]	PULR 05; PVL 4576
*Tropidosuchus romeri*	Arcucci 1990[35]; Dilkes and Arcucci 2012[34]	PVL 4604, 4606
*Euparkeria capensis*	Ewer 1965[36]; Welman 1995[37]; Gower and Weber 1998[38]	SAM-PK 5867 (cast)
*Mystriosuchus westphali*	Hungerbühler and Hunt 2000[39]; Hungerbühler 2002[40]	GPIT 261–001
*Ornithosuchus longidens*	Walker 1964[13]; Baczko and Ezcurra 2013[3]	EM 1, 15, 29; NHMUK PV R 2409, 3142, 3143, 3149, 3562
*Venaticosuchus rusconii*	Bonaparte 1970[7], Baczko et al. 2014[15]	PVL 2578
*Gracilisuchus stipanicicorum*	Romer 1972[41]; Lecuona and Desojo 2012[42]	PURL 08, PVL 4597, 4612
*Turfanosuchus dabaensis*	Young 1973[43]; Wu and Russell 2001[44]	-
*Neoaetosauroides engaeus*	Desojo and Baez 2007[45]	PULR 108, PVL 3525, 4363, 5698
*Longosuchus meadei*	Hunt and Lucas 1990[46]; Parrish 2010[47]	TMM 31185-84A/B
*Stagonolepis robertsoni*	Walker 1961[48]	MCZD 2–4
*Aetosaurus ferratus*	Schoch 2007[49]	SMNS 5770
*Desmatosuchus spurensis*	Parker 2008[50]	TTUP 9024 (cast)
*Quianosuchus mixtus*	Li et al. 2006[51]	-
*Arizonasaurus babbitti*	Nesbitt 2005[52]; Gower and Nesbitt 2010[53]	-
*Shuvosaurus inexpectatus*	Nesbitt and Norell 2006[54]; Lucas et al. 2007[55]; Nesbitt 2011[1]	-
*Effigia okeeffeae*	Nesbitt 2007[56]	-
*Luperosuchus fractus*	Desojo and Arcucci 2009[57], Nesbitt et al. 2013[58]	PULR 04, 057
*Prestosuchus chiniquensis*	Barberena 1978[59]; Mastrantonio et al. 2013[60]	BSPG AS XXV 1-3/5-11/28-41/49, UFRGS PV 0629 T, 156 T
*Saurosuchus galilei*	Alcober 2000[61]	PVL 2062, PVSJ 32
*Batrachotomus kupferzellensis*	Gower 2002[62]	SMNS 52970, 80260–80339
*Fasolasuchus tenax*	Bonaparte 1981[63]	PVL 3850, 3851
*Rauisuchus tiradentes*	Lautenschlager and Rauhut 2014[64]	BSPG AS XXV 60–68, 71–100, 105–119, 121
*Polonosuchus silesiacus*	Sulej 2005[65]; Brusatte et al. 2009[66]	ZPAL Ab III 563
*Postosuchus kirkpatricki*	Weinbaum 2011[67]	-
*Dromicosuchus grallator*	Sues et al. 2003[68]	-
*Hesperosuchus agilis*	Clark et al. 2000[69]	-
*Sphenosuchus acutus*	Walker 1990[70]	-
*Dibothrosuchus elaphros*	Wu and Chatterjee 1993[71]	-
*Litargosuchus leptorhynchus*	Clark and Sues 2002[72]	-
*Kayentasuchus walkeri*	Clark and Sues 2002[72]	-
*Protosuchus haughtoni*	Gow 2000[73]	-
*Alligator mississippiensis*	Brochu 1999[74]	OUVC 9761
*Caiman yacare*	Bona and Desojo 2011[75]	MACN-HE 43694
*Marasuchus lilloensis*	Sereno and Arcucci 1994[76]	-
*Lewisuchus admixtus*	Romer 1972[77]; Bittencourt et al. 2014[78]	PULR 01
*Silesaurus opolensis*	Dzik 2003[79]; Dzik and Sulej 2007[80]	-
*Sacisaurus agudoensis*	Ferigolo and Langer 2007[81]; Langer and Ferigolo 2013[82]	-
*Eoraptor lunensis*	Sereno et al. 1993[83]; 2013[84]	-
*Heterodontosaurus tucki*	Crompton and Charig 1962[85]; Norman et al. 2011[86]	-

#### Skull

The skull of *Riojausuchus tenuisceps* is subtriangular in dorsal view, with a marked constriction on the ventral region of the premaxilla-maxilla contact and a wider constriction between the orbits on the region of the frontals; it has a distinctive overhanging snout that extends anterior to the mandibles (Figs 2 and 3).

The **premaxilla** of *Riojasuchus tenuisceps* is curved anteroventrally (= downturned, [1, 5]) and delimits the external naris on its anterior and posteroventral margins; it can be divided into three parts: a main body, a nasal process, and a maxillary process. The main body of the premaxilla is subrectangular on its anterior end and bears only three teeth (Fig 1A and 1B); this dental configuration is quite uncommon among archosauriforms and can be seen in the archosauriform *Euparkeria capensis* (cast of SAM-PK 5867), the pseudosuchians *Ornithosuchus longidens* (NHMUK PV R 3143), *Gracilisuchus stipanicicorum* (PVL 4597), *Luperosuchus fractus* (PULR 057), *Saurosuchus galilei* (PVSJ 32), and the avemetatarsalian *Heterodontosaurus tucki* [86]. The premaxillary teeth are slightly laterally compressed and curved posteriorly; no serrations can be recognized on any of the preserved teeth (PVL 3827, PVL 3828), probably because the area has been overprepared. The nasal process of the premaxilla is directed posterodorsally and is slightly curved posteriorly; it reaches the anterior process of the nasal at a V-shaped contact. The maxillary process is directed posteriorly; it is apparently laterally overlapped by the ventral process of the nasal on its posterodorsal end and by the maxilla posteriorly. The ventral margin of the maxillary process is edentulous and laterally compressed forming a diastema that holds two hypertrophied dentary teeth when the mandibles occlude, an autapomorphic condition for ornithosuchids (Fig 2). Because of the lateral compression of the premaxilla of *Riojasuchus tenuisceps*, the palatal process is laminar (Fig 2B:Pmx.pp), laterally compressed and ventromedially oriented, and it can be seen in a lateral view, as is also the condition in the archosauriforms *Proterosuchus fergusi* and *Sarmatosuchus otschevi* [87]. Therefore, the maxillary processes of the premaxilla of *Riojasuchus tenuisceps* (PVL 3827, 3828) contact each other along their entire medial surface, differing from the condition of wide-snouted archosaurs such as *Batrachotomus kupferzellensis* (SNMS 52970, 80260) and *Saurosuchus galilei* (PVSJ 32), in which the palatal process is medially directed and the maxillary processes of the premaxilla do not contact each other.

The **maxilla** is almost two times longer than the premaxilla (Table 2); it is anteriorly rounded and bifurcates posteriorly into an ascending process and a posterior process. It does not participate in the delimitation of the external naris (contra Bonaparte 1972[17]) (Fig 2B:Mx) as seen in the majority of archosauriforms (e.g. *Mystriosuchus westphali*: GPIT 261–001, *Ornithosuchus longidens*: NHMUK PV R 3143, *Gracilisuchus stipanicicorum*: PVL 4612, *Saurosuchus galilei*: PVSJ 32, *Luperosuchus fractus*: PULR 057, *Silesaurus opolensis*: [79]), except for most aetosaurs [88], *Effigia okeeffeae* [56] and *Arizonasaurus babbitti* [52] in which the maxilla delimits a small part of this opening. The maxilla forms most of both the antorbital fenestra and antorbital fossa, which are also formed and delimited by the lachrymal posterodorsally and the jugal posteroventrally. The antorbital fenestra is subtriangular, with anterior and ventral acute angles (Fig 2B:AOF), and is surrounded by the nearly semicircular antorbital fossa (Fig 2B:Aofo). This fossa has its anterior and ventral edges almost coinciding with the same edges of the maxilla itself (PVL 3827), unlike most archosaurs in which the ventral margin of the antorbital fossa does not reach the ventral margin of the maxilla (e.g. *Venaticosuchus rusconii*: PVL 2578; *Ornithosuchus longidens*: NHMUK PV R 2409, 3143; *Gracilisuchus stipanicicorum*: PVL 4597; *Neoaetosauroides engaeus*: PVL 5698; *Silesaurus opolensis*: [79]). The lateral surface of the antorbital fossa is bulged at the level of the maxillary teeth alveoli, an uncommon feature; in most archosaurs the lateral surface is smooth. The right maxilla of the holotype of *Riojasuchus tenuisceps* (PVL 3827) has a damaged rounded area on its anterior region caused by poor preparation; on this perforation it is possible to recognize the root of the first maxillary tooth which is twice as long as the tooth crown (Fig 2). No foramina can be recognized on the external surface of the maxilla.

**Table 2:** Measurements of the skulls of PVL 3827 and 3828 (in cm).

	PVL 3827	PVL 3828
Skull length (Pmx-Q)	23.1	25.9
Skull maximum height	8.1	8.0
Premaxilla body length	2.7	3.0
Premaxilla body height	2.2	2.2
Maxilla maximum length	8.2	7.8
Maxilla maximum height	5.6	6.4
Antorbital fenestra length	5.7	4.5
Antorbital fenestra height	3.8	3.7
Nasal length	10.4	-
Lacrimal length	3.2	2.7*
Lacrimal height (exposed in lateral view)	3.9	2.6*
Jugal length	5.2	5.0*
Jugal height	5.8	4.9
Prefrontal length	1.7	*
Prefrontal width	0.3	*
Prefrontal height	1.4	*
Orbit length	3.8	2.4
Orbit height	4.1	2.5*
Frontal length	4.8	5.1
Postorbital length	3.2	*
Postorbital height	3.9	*
Squamosal length	3.7	4.2*
Squamosal height	5.6	*
Infratemporal fenestra length	4.9	2.6*
Infratemporal fenestra height	6.2	4.4*
Supratemporal fenestra length	1.8	*
Supratemporal fenestra width	1.4	*
Quadratojugal height (laterally exposed)	4.4	4.2*
Quadratojugal length at ventral margin	7.5	5*
Quadrate height	6.8	6.1*
Parietal length	5.0	4.7*
Parietals maximum width	4.1	5.3*
Parietals minimum width	1.5	1.8*
Supraoccipital height	1.2*	*
Supraoccipital width	4.4	3.9*
Vomer length	3.5	*
Palatine length	3.2	*
Palatine width	1.8	*
Pterygoid length	10.4	*
Pterygoid width	2.5	*
Ectopterygoid length	3.4*	*
Ectopterygoid width	1.2*	*
Foramen magnum height	0.7	*
Foramen magnum width	1.2	*
Occipital condyle height	1.5	2.0
Occipital condyle width	1.7	2.0
Basal tubera height	2.5	2.7
Basal tubera width at base	3.2	-
Lower jaw length	20.3	20.1
Dentary length	13.4	13.9*
Dentary anterior height	3.0	3.3
Dentary maximum height	4.7	3.7*
Splenial length	14.5*	*
Splenial height	4.6	*
Surangular height	1.3	*
Surangular length	9.0	*
Angular length	7.1*	*
Angular height	1.1	*
External mandibular fenestra length	5.1	*
External mandibular fenestra height	1.2	*

* indicates broken or damaged.

There is no evidence of a palatal process on medial side the maxilla, probably because the area has been overprepared. The ascending process of the maxilla is posterodorsally directed and has the same dorsoventral height along its entire length; it contacts the nasal along its dorsal margin and it is laterally overlapped by the lachrymal on its posterior end. The posterior process of the maxilla tapers posteriorly towards its articulation with the anterior process of the jugal. It differs from loricatans (sensu Nesbitt 2011[1]) such as *Postosuchus kirkpatricki* [30], *Batrachotomus kupferzellensis* (SMNS 52970), and *Saurosuchus galilei* (PVSJ 32) in which the posterior process of the maxilla keeps the same dorsoventral height as the anterior portion of this process, and from aetosaurs in which the posterior process expands dorsoventrally towards its posterior end [1]. Moreover, unlike the condition in most archosaurs, the maxilla does not articulate ventral to the jugal but it apparently overlaps the jugal laterally.

All teeth on the maxillae, as well as the premaxillae, of the holotype of *Riojasuchus tenuisceps* (PVL 3827, Fig 2A) have been damaged during their original preparation and they do not preserve their actual shape; but the referred skull material (PVL 3828, Fig 2C), which we have recently reprepared, has its teeth very well preserved and allow us to provide a detailed description of them. Each maxilla has seven teeth that are laterally compressed and curved posteriorly; both mesial and distal margins are serrated with approximately three denticles per millimeter (Fig 4A). All the maxillary teeth of *Riojasuchus tenuisceps* are even in size and there is no clear evidence of interdental plates between on their medial side (PVL 3827). The CT scan of the holotype allowed us to identify a replacement tooth erupting on the second alveolus of the left maxilla (Fig 4F and 4H: Mrt).

The **nasal** is a slender and anteroventrally oriented element that, in lateral view, bifurcates anteriorly towards its contact with the premaxilla (Figs 2 and 3A:N). Its anterior half is laterally compressed, its posterior half is dorsoventrally depressed and its dorsal surface is flat with no ornamentation (PVL 3827, 3828). The anterior process of the nasal is anteroposteriorly oriented and extends beyond the anterior margin of the maxilla; this process delimits the posterior two-thirds of the dorsal margin of the external naris, and reaches the nasal process of the premaxilla anteriorly at a V-shaped contact. The ventral process of the nasal is anteroventrally directed; it delimits the posterior margin of the external naris, reaching the maxillary process of the premaxilla as in most archosaurs except for stagonolepidid aetosaurs (sensu Desojo et al 2013 [89]), where these two processes do not contact. In *Riojasuchus tenuisceps* (PVL 3827) the ventral ramus of the nasal widens anteriorly unlike most archosaurs, and apparently overlaps the maxillary process of the premaxilla lateroventrally, although this area is slightly damaged; it also contacts the maxilla posteriorly at a straight contact. The posterior process of the nasal reaches the frontal posteriorly by a slightly interdigitated suture, and the lachrymal posterolaterally. It has no lateral process to envelope the lachrymal as seen in eusaurischian dinosaurs and *Eoraptor lunensis* ([1]: ch. 36).

The **lacrimal** is an L-shaped element that delimits the posterodorsal margin of the antorbital fenestra and fossa, and the entire anteroventral margin of the orbit (Figs 2 and 3A). The anterior ramus of the lacrimal of *Riojasuchus tenuisceps* (PVL 3827) is anteroventrally oriented and laterally overlaps the ascending process of the maxilla; it is dorsoventrally high and laterally compressed. The ventral ramus of the lacrimal is posteroventrally directed and laterally overlaps the preorbital process of the jugal. It is almost as high as the orbit and meets the jugal slightly above the ventral margin of the orbit, unlike the condition of sauropodomorphs and theropods in which the lacrimal meets the jugal at the ventral margin of the orbit [1]. The anterior margin of the ventral ramus is expanded anterolaterally, forming a sharp ridge that delimits the posterior margin of the antorbital fossa. This ridge also forms a deep pocket on the anterodorsal end of the fossa (Fig 2:La.p), slightly obscured in lateral view and resembling the condition of theropods and basal saurischians [1:ch. 38]. The morphology differs from pseudosuchians because these do not have a pocket on the lacrimal, although some have a shallow depression on the lateral surface (e.g. *Batrachotomus kupferzellensis*: SMNS 52970, *Aetosaurus ferratus*: SMNS 5770 S16, *Neoaetosauroides engaeus*: PVL 5698).

The **prefrontal** is a laterally compressed element that expands dorsoventrally doubling its height towards its anterior end (Fig 2A and 2B:Prf); it contributes to the anterodorsal margin of the orbit. Because of its lateral compression, the prefrontal is mainly exposed laterally, unlike the condition in most pseudosuchians, in which it is well exposed dorsally as well (e.g. *Ornithosuchus longidens*: NHMUK PV R 3562; *Saurosuchus galilei*: PVSJ 32; *Gracilisuchus stipanicicorum*: PVL 4612). The prefrontal overlaps both the frontal medially and the lachrymal anteriorly, but unlike the morphology in most archosauriforms, the prefrontal does not contact the nasal (Fig 3A:Prf, N). This last feature is only seen in the ornithosuchids *Riojasuchus tenuisceps* (PVL 3827) and *Ornithosuchus longidens* (NHMUK PV R 3562), for in most archosauriforms the nasal and prefrontal contact each other and separate the frontal from the lachrymal [5]. The prefrontal of *Riojasuchus tenuisceps* is smooth on its lateral surface; it has no ventromedial process as that mentioned by Gower and Walker [90] for aetosaurs and crocodylomorphs, and does not contact the palate as that seen in the crocodylomorphs *Dibothrosuchus elaphros* [71], and *Caiman yacare* (MACN-HE 43694). There is no evidence of palpebral bones or areas for articulation with them as those seen on loricatans [91].

The **frontal** of *Riojasuchus tenuisceps* extends along the skull roof from the anterior border of the orbit to the posterior of the same, delimiting the middle region of the dorsal margin of this opening. The frontal is a dorsoventrally depressed and flat bone. It is rectangular in dorsal view, two times longer than wide (Fig 3A:Fr; Table 2), although it has a slight constriction on its lateral margin where the orbit is located. The frontal contacts its counterpart medially but does not form a longitudinal ridge along the midline as seen in the crocodylomorphs *Dromicosuchus grallator* [68], *Hesperosuchus agilis* [69], *Sphenosuchus acutus* [70], and *Dibothrosuchus elaphros* [71], and the loricatans *Batrachotomus kupferzellensis* (SMNS 80260) and *Postosuchus kirkpatricki* [67]. The frontal of *Riojasuchus tenuisceps* contacts the nasal anteriorly at a slightly interdigitated transverse suture, the lachrymal and prefrontal overlap the frontal laterally, the postorbital posterolaterally and the parietal posteriorly. The last two elements contact the frontal by tightly interdigitated sutures. The frontal has the same mediolateral width from its anterior to posterior end, and its external surface is smooth, without ornamentation.

The **postfrontal** is present in *Riojasuchus tenuisceps* (PVL 3827, 3828), unlike dinosaurs, crocodylomorphs, and the poposauroids *Effigia okeeffeae* and *Shuvosaurus inexpectatus* in which lack this element [5, 56, 92]. The postfrontal of *Riojasuchus tenuisceps* is a triangular element in dorsal view that contributes to the delimitation of a small area at the posterodorsal margin of the orbit (Fig 3A:Pof). It contacts the frontal anteromedially and the parietal posteromedially both at tight interdigitated sutures, and the postorbital posterolaterally at a V-shaped suture. It resembles the condition of the basal suchians *Aetosaurus ferratus* (SMNS 5770), *Gracilisuchus stipanicicorum* (PULR 08, PVL 4612) in its triangular shape and dorsal position but differs from the loricatans *Saurosuchus galilei* (PVSJ 32) and *Batrachotomus kupferzellensis* (SMNS 80260) in which the postfrontal is more rectangular and located ventral to the frontal, barely exposed dorsally as a thin strip.

The **postorbital** of *Riojasuchus tenuisceps* is a triradiate element, which can be divided into anterior, ventral, and posterior processes. The anterior process is anteromedially projected; it contacts the postfrontal anterodorsally at a V-shaped suture, and delimits the anterolateral margin of the supratemporal fenestra (Figs 2 and 3A:STF). The ventral process of the postorbital of *Riojasuchus tenuisceps* (PVL 3827, 3828) is posterodorsally oriented at about 65° from the horizontal (Fig 2:Po), which is a slightly more vertical orientation than that seen in pseudosuchians such as *Ornithosuchus longidens* (NHMUK PV R 2409: 55°) and *Aetosaurus ferratus* (SMNS 5770: 55°) even more vertical than that of *Gracilisuchus stipanicicorum* (PULR 08: 50°) and *Saurosuchus galilei* (PVSJ 32: 40°). This process tapers and overlaps the jugal anterolaterally and delimits most of the posterior margin of the orbit and the anterior margin of the dorsal half of the infratemporal fenestra. The posterior process of the postorbital extends posteriorly to contact the squamosal at a V-shaped suture; it delimits the dorsal margin of the infratemporal fenestra and the lateral margin of the supratemporal fenestra (Fig 2:ITF, STF). The supratemporal fenestrae of *Riojasuchus tenuisceps* (PVL 3827, 3828) face dorsally and are reduced to about one quarter of the infratemporal fenestra and one third of the orbit.

The **parietal** of *Riojasuchus tenuisceps* (PVL 3827) is not fused to its counterpart, as is the case of most archosauriforms except for crocodylomorphs (where both parietals are completely fused to each other). In *Riojasuchus tenuisceps*, the parietals are not ornamented and rectangular, being anteroposteriorly elongated and barely longer than the frontals (Fig 3A:Pa; Table 2). Each parietal bears a divergent posterolateral process on its posterior end that gives a V-shape to the occipital margin, resembling the condition of most archosauriforms, but differing from crocodylomorphs (e.g. *Sphenosuchus acutus* [70], *Dibothrosuchus elaphros* [71], *Protosuchus haughtoni* [73], and *Caiman yacare*: MACN-HE 43694), where the occipital margin of the parietals is straight. The parietal is sutured anteriorly by a tight interdigitation with the frontal and postfrontal; it contacts the postorbital and the squamosal laterally; it is tightly sutured to the supraoccipital posteromedially and reaches the paroccipital processes of the opisthotics posteriorly. There is a noticeable change of slope at the midpoint of the parietals of *Riojasuchus tenuisceps* (PVL 3827, 3828) with the anterior half of the parietal anteroventrally oriented and the posterior half posteroventrally oriented (Fig 2B:Pa). The posterolateral process of the parietal of *Riojasuchus tenuisceps* slopes posteroventrally nearly at 45 degrees as seen only in *Ornithosuchus longidens* (NHMUK PV R 2409) and aetosaurs (e.g. *Neoaetosauroides engaeus*: PVL 5698), and differing from a more vertical condition seen in most archosaurs [1, 5]. The parietal delimits the medial margin of the supratemporal fenestra, the anterior margin of the posttemporal fenestra, and it does not form a supratemporal fossa as is the condition of most basal archosauriforms.

The **jugal** of *Riojasuchus tenuisceps* has a distinctive shape not seen in other archosaurs, except for *Venaticosuchus rusconii*; it is a triradiate element but its ascending process bifurcates dorsally. The jugal delimits the antorbital fenestra posteriorly, the orbit ventrally, and the infratemporal fenestra anteroventrally (Fig 2A–2C:J). As mentioned, the jugal of *Riojasuchus tenuisceps* participates in the margin of the antorbital fenestra, a condition seen also in the archosauriform *Proterosuchus fergusi* [26], phytosaurs, and some proterochampsids (*Chanaresuchus bonapartei*: PVL 4575, 4586; *Gualosuchus reigi*: PVL 4576), the pseudosuchians *Ornithosuchus longidens* (NHMUK PV R 2409, 3142), *Venaticosuchus rusconii* (PVL 2578), and *Gracilisuchus stipanicicorum* (PVL 4598), sauropodomorphs and ornithischians. This differentiates *Riojasuchus tenuisceps* from crocodylomorphs, *Revueltosaurus callenderi*, some aetosaurs and loricatans, and theropods in which the jugal is excluded from the margin of this fenestra by either the lachrymal or the maxilla [1]. The jugal of *Riojasuchus tenuisceps* (PVL 3827) also forms the posteroventral region of the antorbital fossa as seen in *Venaticosuchus rusconii* (PVL 2578) and *Ornithosuchus longidens* (NHMUK PV R 3142), but differing from the condition in most pseudosuchians (e.g. *Neoaetosauroides engaeus*: PVL 5698; *Batrachotomus kupferzellensis*: SMNS 52970; *Saurosuchus galilei*: PVL 2062; *Sphenosuchus acutus*: [70]) and avemetatarsalians (e.g. *Heterodontosaurus tucki*: [86]; *Herrerasaurus ischigualastensis*: [93])

The anterior process of the jugal is short; anteroposteriorly directed and has a slightly wavy sutural line with the posterior process of the maxilla, where the jugal slightly widens dorsoventrally. The ascending process has a wide vertical bony strut that separates the antorbital fenestra from the infratemporal fenestra [4] (Fig 2B:J.vp); this is an unusual condition because in most archosauriforms the ascending process is slender, posterodorsally directed and separates the orbit from the infratemporal fenestra (e.g., *Chanaresuchus bonapartei*: PVL 4575, 4586; *Ornithosuchus longidens*: NHMUK PV R 2409; *Gracilisuchus stipanicicorum*: PVL 4612; *Neoaetosauroides engaeus*: PVL 4363; *Batrachotomus kupferzellensis*: SMNS 52970; *Protosuchus haughtoni*: [73]). This vertical strut elevates the orbit and bifurcates dorsally into a preorbital ramus and a postorbital ramus, both of which taper dorsally. This gives the orbit a distinctive V-shaped ventral margin (PVL 3827, 3828) [1] (Fig 2:J). The posterior process of the jugal is three times longer than the anterior process and it is anteroposteriorly oriented. It dorsally overlaps the anterior process of the quadratojugal as in the basal archosauriforms *Erythrosuchus africanus* [28], *Euparkeria capensis* [36], *Chanaresuchus bonapartei* (PVL 4575, 4586), the poposaurids *Quianosuchus mixtus* [51] and *Arizonasaurus babbitti* [52], and the loricatans *Batrachotomus kupferzellensis* (SMNS 52970) and *Prestosuchus chiniquensis* (UFRGS-PV 156 T). The jugal of *Riojasuchus tenuisceps* (PVL 3827, 3828) has an anteroposteriorly short lateral protuberance on the ventral margin of its central region (Fig 2B:J.pb). It differs from the ridge seen on the lateral surface of the jugal of *Ornithosuchus longidens* (NHMUK PV R 3142) but slightly resembles the protuberance seen in *Garjainia madiba* [29], although it is not so expanded laterally.

The **quadratojugal** is an L-shaped element that delimits a portion of the posterior margin of the infratemporal fenestra (less than 80% of its posterior margin as most archosaurs except for some aetosaurs, “rauisuchids” and crocodylomorphs) and the lateral margin of the quadrate foramen. The anterior process of the quadratojugal of *Riojasuchus tenuisceps* is anteroposteriorly directed and laterally overlapped by the jugal; the dorsal process of the quadratojugal is anterodorsally oriented, dorsally overlapped by the squamosal, and contacts the quadrate medially all along its medial margin (Fig 2B:Qj). The anterodorsal direction of this dorsal process gives this fenestra a distinctive L-shape otherwise seen only in *Ornithosuchus longidens* (Fig 2B:ITF). The quadratojugal of *Riojasuchus tenuisceps* (PVL 3827) resembles that of some proterochampsids such as *Chanaresuchus bonapartei* (PVL 4586) and *Gualosuchus reigi* (PULR 05) because of the presence of a concavity on its anterior margin. Nonetheless, it differs from the later because, as in most archosauriforms, it has a smooth lateral surface without a temporal fossa and therefore without a ridge delimiting it, as seen in *Chanaresuchus bonapartei* (PVL 4586) and *Tropidosuchus romeri* (PVL 4606). The quadratojugal of *Riojasuchus tenuisceps* also delimits the lateral margin of the quadrate foramen (Fig 5:Q.f).

The **squamosal** is a tetraradiate element that can be divided into anterior, ventral, posterior and medial processes. The anterior process is very short and articulates with the posterior process of the postorbital, separating the supratemporal from the infratemporal fenestra (Figs 2 and 3:Po, STF, ITF). The ventral process of the squamosal is ventrally directed unlike that of aetosaurs which has a marked anteroventral orientation. This ventral process in *Riojasuchus tenuisceps* reaches the quadratojugal ventrally and gives the infratemporal fenestra a distinctive L-shape, also seen in *Ornithosuchus longidens* (NHMUK PV R 2409). This process has a great participation on the margin of the infratemporal fenestra, delimiting almost half of its posterior margin (Fig 2:ITF); this condition resembles that of most archosaurs excepting aetosaurs in which the squamosal barely participates on the margin of this fenestra (e.g. *Neoaetosauroides engaeus*: PVL 4363) or is excluded from it by the quadratojugal and postorbital (e.g. *Aetosaurus ferratus*: SMNS 5770). The posterior process of the squamosal of *Riojasuchus tenuisceps* is hook-like and extends posterior to the dorsal head of the quadrate resembling that of most archosauriforms, except for *Proterosuchus fergusi* [26] and *Erythrosuchus africanus* [28]. It laterally overlaps the opisthotic forming the paroccipital processes (Fig 2:Pp). The lateral surface of the squamosal of *Riojasuchus tenuisceps* does not have any ridge like that seen on the basal loricatans *Saurosuchus galilei* (PVL 2062), *Prestosuchus chiniquensis* (UFRGS-PV 156 T), and *Batrachotomus kupferzellensis* (SMNS 52970). Furthermore, the squamosal of *Riojasuchus tenuisceps* lack ridges delimiting asupratemporal fossa on its dorsal surface as that seen on *Postosuchus kirkpatricki* [67], *Polonosuchus sileasicus* (ZPAL Ab III 563), *Rauisuchus tiradentes* (BSPG AS XXV 62), and *Batrachotomus kupferzellensis* (SMNS 52970, 80260). The medial process of the squamosal of *Riojasuchus tenuisceps* is not exposed on any of the skulls (PVL 3827, 3828) because they are completely articulated.

The **quadrate** is a dorsoventrally elongated bone, posteroventrally directed as in most archosaurs with the exception of aetosaurs (e.g. *Neoaetosauroides engaeus* PVL 5698), *Shuvosaurus inexpectatus*, spinosaurids and ornithomimids in which the quadrate is anteroventrally oriented (Fig 2B:Q). The quadrate of *Riojasuchus tenuisceps* delimits the dorsal, medial, and ventral margins of the quadrate-quadratojugal foramen, which is formed in between the lateral contact of the quadrate with the quadratojugal (Fig 5:Q.f). The quadrate contacts the squamosal laterodorsally along its dorsal half, the opisthotic dorsally, and overlaps the pterygoid anteromedially. The dorsal head of the quadrate of *Riojasuchus tenuisceps* (PVL 3827) is expanded anteromedially; it is partially exposed laterally as seen in a wide variety of dinosaurs and pseudosuchians (e.g. *Turfanosuchus dabaensis* [44], *Quianosuchus mixtus* [51], *Saurosuchus galilei*: PVSJ 32, *Aetosaurus ferratus*: SMNS 5770), excepting crocodylomorphs, where the squamosal covers the entire head of the quadrate [1]. The dorsal head of the quadrate fits into a concavity formed by the squamosal and the paroccipital process, apparently forming the synovial otic joint as is the condition in most archosaurs, excepting thyreophoran dinosaurs, which have akinetic skulls [94]. The quadrate has a dorsoventrally oriented ridge on its posterior surface (Fig 5:Q.r). Unlike *Postosuchus kirkpatricki* [67] and *Polonosuchus sileasicus* (ZPAL Ab III 563), where the ridge is ventral to the quadrate-quadratojugal foramen, in *Riojasuchus tenuisceps* the ridge is dorsolateral to the foramen. The distal end of the quadrate is divided by a shallow groove, resulting in two small condyles. The medial condyle is rounded whereas the lateral condyle is slightly compressed anteroposteriorly They articulate with the concave articular surfaces of the articular from the lower jaws.

#### Palatal complex

The **vomer** of *Riojasuchus tenuisceps* is a laterally compressed and anteroposteriorly directed element that is located between the choanae, delimiting their medial margins, and forming the anterior end of the palate (Fig 3B:V). Each vomer contacts its counterpart medially, the premaxilla anteriorly, and the palatine posteriorly. There is no evidence of teeth on the ventral surface of the vomers of *Riojasuchus tenuisceps*, unlike proterochampsians (e.g. *Tropidosuchus romeri*: PVL 4601) and proterosuchids (e.g. *Proterosuchus fergusi* [26]), which have teeth along their vomers.

The **palatine** contacts the vomer anteriorly, the pterygoid medially and posteriorly, and the maxilla laterally. It delimits the posterior margin of the choana but has no defined fossa around the choana as is the case of most archosauriforms (Fig 3B:Pl, Ch), except for the crocodylomorphs *Sphenosuchus acutus* [70], *Dibothrosuchus elaphros* [71], and *Kayentasuchus walkeri* [72], which have a rim delimiting a fossa around the choana on the ventral surface of the palatine. The palatine of *Riojasuchus tenuisceps* (PVL 3827) has an anterolaterally directed ridge running across its ventral surface (Fig 3B:Pl.r). It has no palatine teeth, like most archosaurs but differing from non-archosaurian archosauriforms such as *Chanaresuchus bonapartei* (PULR 07), *Tropidosuchus romeri* (PVL 4601), and *Doswellia kaltenbachi* [31].

The **pterygoid** is the largest element of the palate (Fig 3B:Pt; Table 2). It contacts the vomer anteriorly through the tip of its anteromedial process, the palatines anterolaterally, and the ectopterygoids ventrolaterally; the pterygoid overlaps the quadrate posterolaterally through the quadrate process, and reaches the basipterygoid processes of the basisphenoid posteriorly. It only contacts the other pterygoid medially on its anterior-most end, but most of the pterygoid does not contact its counterpart, forming an interpterygoid vacuity (Fig 3B:Iptv) similar to that of *Euparkeria capensis* [36] and *Turfanosuchus dabaensis* [44]. *Riojasuchus tenuisceps* (PVL 3827) differs from *Ornithosuchus longidens* (NHMUK PV R 2409), because on the latter the pterygoids do contact each other at the midline along their entire medial margin and therefore do not form an interpterygoid vacuity. The palatal process of the pterygoid of *Riojasuchus tenuisceps* is smooth and has no teeth as is the case of most archosaurs. This contrasts with the condition in the gracilisuchid *Turfanosuchus dabaensis* [44] and the theropod dinosaur *Eoraptor lunensis* [84] which have pterygoid teeth, and many archosauriforms such as proterochampsids, doswellids, and proterosuchids. The palatal process delimits the palatine-pterygoid fenestra medially and posteriorly, and the suborbital fenestra posteromedially (Fig 3B:Pl-Pt.f, Sor.f). The presence of the palatine-pterygoid fenestra is a synapomorphy of Ornithosuchidae and therefore has only been registered in *Ornithosuchus longidens*, *Riojasuchus tenuisceps*, and *Venaticosuchus rusconii* [4, 5]. The quadrate process of the pterygoid, which contacts both the quadrate and the basipterygoid process, has a concave surface where the basipterygoid process fits as a peg and socket articulation, constituting the basal cranial joint, and a posterolateral laminar projection that laterally overlaps the pterygoid process of the quadrate.

The **ectopterygoid** is a comma-shaped element that contacts the jugal ventrolaterally and the pterygoid dorsomedially (Fig 3B:Ept). The ventral disposition of the ectopterygoid of *Riojasuchus tenuisceps* resembles that of basal archosauriforms and pseudosuchians, and differs from dinosaurs, where the ectopterygoid is located dorsal to the pterygoid. The ectopterygoid of *Riojasuchus tenuisceps* delimits the posterior margin of the suborbital fenestra; this fenestra is equal in size to the palatine-pterygoid fenestra (Fig 3B:Pl-Pt.f, Sor.f; Table 1), unlike that of *Ornithosuchus longidens*, in which the suborbital fenestra is about three times larger than the palatine-pterygoid fenestra [13]. On the other hand, the suborbital fenestra of *Riojasuchus tenuisceps* is proportionally smaller (fenestra/skull length: 1/20) than that of other archosauriforms such as *Ornithosuchus longidens* (1/11), *Neoaetosauroides engaeus* (1/7.2), *Saurosuchus galilei* (1/7.5), *Chañaresuchus bonapartei* (1/11.4).

#### Braincase

The **supraoccipital** is an element located in the posteriormost area of the skull roof, appearing subtriangular in posterodorsal view (Fig 3A:So). It forms the dorsal margin of the foramen magnum as seen in most archosauriforms (Fig 5:FM), excepting proterosuchids and erythrosuchids in which the exoccipitals exclude the supraoccipital from this foramen. The supraoccipital also delimits the medial margins of the posttemporal opening (PVL 3827; Fig 3A:PTF). In *Riojasuchus tenuisceps*, as well as phytosaurs, aetosaurs, and the suchians *Gracilisuchus stipanicicorum* (PULR 08) and *Saurosuchus galilei* (PVSJ 32), the posttemporal opening is slightly wider than half the diameter of the foramen magnum. This is unlike that of proterochampsids and dinosaurs in which this fenestra is reduced, or crocodylomorphs in which this opening is absent. Although the sutures are difficult to trace, the supraoccipital contacts the parietal anteriorly at a tight interdigitated suture, and contacts the ophistotic posterolaterally at a straight suture. The contact with the exoccipital is unclear because of the poor preservation of that region. The external dorsal surface of the supraoccipital of *Riojasuchus tenuisceps* is smooth. There is no evidence of any ridge at the midline for the insertion of nuchal muscles unlike the condition seen in the pseudosuchians *Neoaetosauroides engaeus* (PVL 5698), *Arizonasaurus babbitti* [53], *Saurosuchus galilei* [61], and *Batrachotomus kupferzellensis* (SMNS 80260), or anterolateral ridges as those seen in the dinosauriforms *Silesaurus opolensis* [79] and *Lewisuchus admixtus* (PULR 01).

The **ophistotic** is mediolaterally elongated; posterolaterally oriented. It forms, along with the squamosal, the paroccipital process which has a barely expanded distal end (Figs 3A and 5:Op), as seen in phytosaurs, aetosaurs (e.g. *Neoaetosauroides engaeus*: PVL 5698), *Gracilisuchus stipanicicorum* (PULR 08; PVL 4612), *Batrachotomus kupferzellensis* (SMNS 80260), and *Saurosuchus galilei* [1, 61]. This contrasts with the condition of *Postosuchus kirkpatricki* [67] and some crocodylomorphs (e.g. *Sphenosuchus acutus* [70]), where the distal end of the paroccipital process expands dorsally [1]. The ophistotic of *Riojasuchus tenuisceps* is apparently fused medially to the exoccipital (Fig 5:Op, Eo); the ventral ramus of the opisthotic extends lateral to the exoccipital area, and the sutures between these two elements cannot be seen, a condition common in most archosauriforms. The ophistotic contacts the parietal anteriorly at a straight suture, overlaps the squamosal laterally, the quadrate ventrally, the basioccipital posteromedially, and reaches the supraoccipital medially. The ophistotic of *Riojasuchus tenuisceps* delimits the posterior margin of the post-temporal fenestra and extends laterally beyond the supratemporal fenestra. This resembles the condition of most archosaurs (Fig 3A:Op, PTF, STF), but is unlike that seen in *Gracilisuchus stipanicicorum* [41] and some crocodylomorphs (e.g. *Protosuchus haughtoni* [73], *Litargosuchus leptorhynchus* [72]), in which the lateral extent of the paroccipital process is at the margin or medial to the supratemporal fenestra. The external surface of the ophistotic is smooth to slightly striated (PVL 3828) but does not form a marked subhorizontal ridge as seen in the loricatans *Batrachotomus kupferzellensis* (SMNS 80260) and *Saurosuchus galilei* (PVSJ 32).

The **exoccipital** is poorly preserved on both skulls (PVL 3827, 3828). The exoccipital-opisthotic suture line is not marked, as occurs in many basal archosauriforms [95]. Nevertheless, the exoccipital is here identified as the dorsolaterally oriented pillar that contacts the basioccipital and the ophistotic region (Fig 5:Eo). The exoccipitals of *Riojasuchus tenuisceps* do not contribute to the occipital condyle, resembling the condition of *Euparkeria capensis* [38]. This condition differs from most archosauriforms in which the occipital condyle is composed not only of the basioccipital but also the exoccipitals (e.g. *Pseudochampsa ischigualastensis*: [96]; *Neoaetosauroides engaeus*: PLV 5698; *Saurosuchus galilei*: PVSJ 32; and *Arizonasaurus babbitti*: [53]). The exoccipitals of *Riojasuchus tenuisceps* do not contact each other medially as seen in *Effigia okeeffeae*, *Shuvosaurus inexpectatus* and crocodylomorphs [1], but only delimit the lateral margins of the foramen magnum (Fig 5:FM). This constrasts the condition seen in most archosaurs, where the exoccipitals meet at the midline excluding the basioccipital from the foramen magnum, and therefore delimiting this opening ventrally. The exoccipitals of *Riojasuchus tenuisceps* (PVL 3827, 3828) have no evidence of a lateral exoccipital ridge as seen in *Batrachotomus kupferzellensis* (SMNS 90042) and *Stagonolepis robertsoni* (MCZD 2–4). Apossible foramen for the cranial nerve XII on the right exoccipital of the holotype of *Riojasuchus tenuisceps* is visible but should be considered carefully because of the poor preservation of this region (Fig 5:CN XII?).

The **basioccipital** forms all the occipital condyle and the posterodorsal portion of the basal tubera (Fig 5:Bo, Bt); it contacts the basisphenoid anteroventrally at a straight suture and the exoccipitals dorsolaterally. The occipital condyle is almost spherical and does not have evidence of a notochordal pit, contrasting with that seen on *Batrachotomus kupferzellensis* (SMNS 80260), *Saurosuchus galilei* (PVSJ 32), and *Doswellia kaltenbachi* [31]. The occipital condyle lies very close to the posterior side of the basal tubera and therefore the basioccipital has a poor development of the condylar neck (Fig 3B:Bo) as seen in *Batrachotomus kupferzellensis* (SMNS 80260), *Postosuchus kirkpatricki* [67], *Saurosuchus galilei* (PVSJ 32), and *Doswellia kaltenbachi* [31]. However, it does not reach the reduction seen in *Archeopelta arborensis* (CPEZ-239a), *Euparkeria capensis* (cast of SAM-PK 5867), *Proterosuchus fergusi* [26], and *Marasuchus lilloensis* [97] where the condylar neck is completely absent. The basal tubera of the basioccipital is a bilobed structure in which each lobe is separated from the other by a shallow ventromedial notch. Each lobe is located ventrolateral to the occipital condyle (Fig 5:Bt); they contact the exoccipital dorsally and the basal tubera of the parabasisphenoid anteriorly. On the posteroventral surface of the basal tubera of the basioccipital, where both lobes meet medially, the basioccipital has an anteroposteriorly compressed depression, the basioccipital recess (Fig 3B:Bo.r), resembling the condition of most archosaurs excepting the rauisuchid *Postosuchus kirkpatricki* [67] which lacks this depression. The presence of this basioccipital recess also distinguishes *Riojasuchus tenuiceps* from proterochampsids and erytrhosuchids which do not have this structure.

The **parabasisphenoid** forms the anterior part of the basal tubera and the basipterygoid processes (Fig 3B:Bs, Bpt), and it is anteroventrally directed so that the basipterygoid processes are located anteroventrally to the basal tubera as in most archosauriforms (e.g. *Chanaresuchus bonapartei*: PVL 4586, *Euparkeria capensis*: cast of SAM-PK 5867, *Neoaetosauroides engaeus*: PVL 5698, *Saurosuchus galilei*: PVSJ 32, *Arizonasaurus babbitti*: [52], *Silesaurus opolensis*: [79]). This configuration contrasts with that seen in *Proterosuchus fergusi*, where the basal tubera and the base of the basipterygoid processes are at the same level. The basisphenoid recess (= median pharyngeal recess) is located on the ventromedial region of the parabasisphenoid (Fig 3B:Bs.r), between the basipterygoid processes, and it is shallow like that seen in aetosaurs (e.g. *Neoaetosauroides engaeus*: PVL 5698, *Desmatosuchus spurensis*: cast of TTUP 9024, *Aetosaurus ferratus*: SMNS 5770) [1]. *Riojasuchus tenuisceps* (PVL 3827, 3828) lacks an intertuberal plate between the basioccipital recess and the basisphenoid recess as in most archosauriforms (e.g. phytosaurs, aetosaurs, “rauisuchians”), excepting proterosuchids, which have this structure separating both recesses [95](e.g. *Proterosuchus fergusi* [95], *Garjainia madiba* [29]). As in basal archosauriforms and pseudosuchians, the lateral surface of the parabasisphenoid of *Riojasuchus tenuisceps* lacks an anterior tympanic recess (PVL 3827), that structure can be identified only in neotheropods [97] and some basal dinosauromorphs [1].

The basipterygoid processes of *Riojasuchus tenuisceps* are not enlarged like that of non-crocodyliform crocodylomorphs but are of a moderate size compared to the tubera, as is the condition of most archosauriforms (Fig 3B:Bpt). The basipterygoid processes are posteriorly oriented and diamond-shaped, with a marked notch that separates them from the basal tubera, and contact the pterygoid at an apparently non-sutural articulation. They resemble those seen in *Postosuchus kirkpatricki* [67], *Batrachotomus kupferzellensis* [62], *Saurosuchus galilei* (PVSJ 32), *Sphenosuchus acutus* [70], aetosaurs and phytosaurs, but differ from those of *Chanaresuchus bonapartei* (PULR 07), *Effigia okeeffeae* [56], *Shuvosaurus inexpectatus* [1], *Silesaurus opolensis* [79], and *Marasuchus lilloensis* [76] in which these processes face more anteriorly [1]. The cultriform process is difficult to identify, but extends anteriorly between the dorsomedial margins of the pterygoids. The foramina for the exit of the internal carotids cannot be recognized on PVL 3827 or PVL 3828. Nonetheless, we can eliminate the posibility that these openings are located on the ventral surface of the parabasisphenoid, for that area is well preserved and there is no evidence of these foramina there (Fig 3B:Bs).

The **prootic** and **laterosphenoid** cannot be clearly recognized on the holotype (PVL 3827) because of the poor preparation of that region, but they are exposed on the specimen PVL 3828, which we have recently reprepared. Nevertheless, they are poorly preserved and damaged by the crushing of the skull roof. These elements form the lateral wall of the braincase, with the laterosphenoid contacting the frontal dorsally and the prootic posteriorly (Fig 2B:Pr, Ls). On the middle region of the lateral side of the braincase of PVL 3828 there is a round pit that could correspond to the foramen for the trigeminal nerve (cranial nerve V), which would also indicate the prootic-laterosphenoid suture (Fig 2B:CN V).

#### Lower jaw

The **dentary** of *Riojasuchus tenuiceps* is anteroposteriorly elongated, dorsoventrally low, and it bifurcates posteriorly, occupying more than half the length of the lower jaw (Fig 6:De; Table 2). The anterior end of the dentary contacts its counterpart medially along a class I mandibular symphysis (flat, smooth and equally high as deep surface [98]); this area is ventrally expanded compared to the rest of the dentary. The anterior end of the dentary is rounded as in most archosauriforms, unlike that of aetosaurs (e.g. *Neoaetosauroides engaeus*: PVL 3525, *Aetosaurus ferratus*: SMNS 5770) and some silesaurid dinosauriforms (e.g. *Silesaurus opolensis*, *Sacisaurus agudoensis*) [1], in which the dentary is pointed. The lateral surface of the dentary is smooth and no foramina can be recognized either on PVL 3827 or PVL 3828. The dorsal surangular process contacts the surangular but the type of contact is unclear because of the poor preservation of this region; the dorsal surangular process of the dentary delimits the anterodorsal margin of the external mandibular fenestra (Fig 6A and 6B: EMF). The ventral angular process of the dentary tapers posteriorly, overlaps the angular, and delimits the anteroventral margin of the external mandibular fenestra. The dentary has nine teeth along its dorsal margin, but no interdental plates can be recognized. The first tooth is broken but small in section (Fig 4B), whereas the second and third teeth are hypertrophied, being three times larger than the posterior ones, slightly curved posteriorly, and have no serrations preserved on their margins, probably because of preparation (PVL 3827, 3828). These teeth are located on the anterior region of the dentary and, when the mandibles occlude, they fit in the edentulous diastema of the premaxilla. CT scan images allowed us to see the long posteroventrally curved roots of this hypertrophied teeth, which are twice as tall as the crown, and a replacement tooth on the third alveolus of the dentary (Fig 4E and 4G:Al, Drt). The posterior teeth are much smaller and some of them are barely curved posteriorly. One tooth from the posterior region of the dentary was exposed while repreparing the lower jaws of PVL 3828, and it has serrations on the distal margin (Fig 4C). The posterior teeth of the holotype (PVL 3827), which have been overprepared and lost all trace of the presence of denticles. Contra Baczko et al. 2014 [4], the dental configuration in *Riojasuchus tenuisceps* (PVL 3827, 3828) resembles that of *Ornithosuchus longidens* (NHMUK PV R 3143), and differs from *Venaticosuchus rusconii* (PVL 2578), in having a first small tooth before the hypertrophied caniniform teeth on the anterior region of the dentary.

The **splenial** of *Riojasuchus tenuisceps* covers the Meckelian groove medially for there is no visible exit on either preserved hemimandible of PVL 3827, which are disarticulated and have their sypmphyseal articular surfaces exposed (Fig 6C and 6D:Sp). The anterior end of the splenial of *Riojasuchus tenuisceps* (PVL 3827) reaches the level of the third dentary teeth. Because of the anteroposteriorly expanded symphysis of the mandibles the splenial has an important participation in this suture as also seen in phytosaurs [5] and the pseudosuchians *Ornithosuchus longidens*, *Shuvosaurus inexpectatus*, and *Effigia okeeffeae* [56]. *Riojasuchus tenuisceps* (PVL 3827, 3828) has no mylohyoid foramen on the ventral surface of the splenial as is the condition for all pseudosuchians; the mylohyoid foramen has only been recognized in saurischian dinosaurs [1].

The **surangular** is an anteroposteriorly directed and elongated element; it forms the dorsal margin of the posterior half of the mandible, and delimits the posterodorsal margin of the external and internal mandibular fenestrae (Fig 6:Sa, EMF, IMF). The surangular has a very well developed surangular shelf on its dorsal margin (Fig 6B:Sa.sh), sharper than that seen in *Ornithosuchus longidens* (NHMUK PV R 2409) and *Gracilisuchus stipanicicorum* (PVL 4597). A deep, rounded foramen can be clearly recognized close to the posterior end of the surangular (Fig 6B:Sa.f). The surangular foramen in *Riojasuchus tenuisceps* is small, like that of most archosauriforms (e.g. *Chanaresuchus bonapartei*: PVL 4676, *Euparkeria capensis*: cast of SAM-PK 5867, *Ornithosuchus longidens*: NHMUK PV R 3142, *Aetosaurus ferratus*: SMNS 5770), differing from the poposauriods *Effigia okeeffeae* and *Shuvosaurus inexpectatus* [56] in which the surangular foramen is expanded, and also from crocodylomorphs (e.g. *Sphenosuchus acutus* [70], *Dibothrosuchus elaphros* [71]) in which the surangular foramen is completely absent (e.g. *Caiman yacare*: MACN-HE 43694).

The **angular** of *Riojasuchus tenuisceps* is a laterally compressed and slender element that delimits the ventral margin of the external mandibular fenestra (Fig 6:An, EMF). The angular (PVL 3827; Fig 6) forms the complete ventral margin of the external mandibular fenestra as seen in the archosauriforms *Proterosuchus fergusi* [25] and *Erythrosuchus africanus* [28], phytosaurs, the ornithosuchid *Venaticosuchus rusconii* (PVL 2578), gracilisuchids, and the shuvosaurid *Effigia okeeffeae* [56]. This condition differs from that of proterochampsids, *Euparkeria capensis* (cast of SAM-PK 5867), most aetosaurs (excepting *Aetosaurus ferratus*: SMNS 5770), and loricatans in which the angular delimits the posterior three quarters of the external mandibular fenestra. The angular of *Riojasuchus tenuisceps* tapers anteriorly, contacting the dentary at an oblique suture. Tha angular also overlaps the prearticular medially, reaches the surangular dorsally, and contacts the articular posteriorly at a straight contact. The angular is mainly exposed in lateral view but also reaches the ventral margin of the hemimandible in *Riojasuchus tenuisceps* (PVL 3827, 3828) (Fig 6D:Sa); this resembles the condition in other pseudosuchians such as the aetosaur *Neoaetosauroides engaeus* (PVL 4363) and the loricatan *Batrachotomus kupferzellensis* (SMNS 80260). The external surface of the angular is convex and smooth unlike the rugose surface seen in the pseudosuchians *Ornithosuchus longidens* [13] and *Postosuchus kirkpatricki* [67].

The **prearticular** of *Riojasuchus tenuisceps* forms the posteroventral margin of the lower jaw and delimits the ventral margin of the internal mandibular fenestra (Fig 6D:Pra, IMF). The prearticular (PVL 3827) is exposed medially and ventrally, unlike that of the aetosaur *Neoaetosauroides engaeus* (PVL 4363), and the loricatans *Batrachotomus kupferzellensis* (SMNS 80260) and *Postosuchus kirkpatricki* [67], where the element is only exposed medially and its ventral surface is covered by the angular. The prearticular of *Riojasuchus tenuisceps* is slender and anteroposteriorly elongated; its anterior region curves anterodorsally and its posterior region bifurcates posteriorly into a posterior process and a medial one. The anterior projection of the prearticular is medially overlapped by the splenial and ventrolaterally overlapped by the angular. The posterior half of the prearticular of *Riojasuchus tenuisceps* (PVL 3827, 3828) is low and curves anterodorsally but it does not expand dorsoventrally, contrasting with the fan-shaped portion seen in the loricatans *Batrachotomus kupferzellensis* (SMNS 80260), *Postosuchus kirkpatricki* [67], and *Rauisuchus tiradentes* (BSPG AS XXV 68). The posterior process of the prearticular of *Riojasuchus tenuisceps* covers the surangular laterally and the articular posterodorsally, and the medial projection of the prearticular contacts the articular on its posterodorsal end.

The **articular** is triangular in dorsal view, with a glenoid fossa that bears two cotyles separated by a delicate transverse rim where the distal condyles of the quadrate articulate. The medial cotylus is larger than the lateral (PVL 3827) (Fig 6D:Ar, Gl). The glenoid fossa of *Riojasuchus tenuisceps* is located at the level of the dorsal margin of the mandible, unlike the condition in basal archosauriforms, aetosaurs, ornithischians and sauropodomorphs, in which the glenoid is at a lower level below the dorsal margin of the mandible [1]. The articular is overlapped by the surangular anterolaterally and the prearticular anteromedially and ventrally. On its posterior end, the articular of *Riojasuchus tenuisceps* has a retroarticular process that is 1.5 times the anteroposterior length of the glenoid fossa (PVL 3827) (Fig 6:Ar.rp). As seen in most archosauriforms, the articular of *Riojasuchus tenuisceps* is concave posterior to the glenoid fossa and lacks any dorsomedial projection in that area differing from the condition in loricatans (e.g. *Postosuchus kirkpatricki* [67], *Batrachotomus kupferzellensis*: SMNS 80260, *Fasolasuchus tenax*: PVL 3850), the aetosaurs *Stagonolepis robertsoni* and *Longosuchus meadei* [47], and basal crocodylomorphs (e.g. *Hesperosuchus agilis* [69], *Dromicosuchus grallator* [68], *Protosuchus haughtoni* [73]), which have a dorsomedial projection. Moreover, the ventromedial process (sensu Nesbitt 2011 [1]) of the articular seen in various phytosaurs and paracrocodylomorphs (e.g *Postosuchus kirkpatricki* [67], *Batrachotomus kupferzellensis*: SMNS 80260, *Fasolasuchus tenax*: PVL 3850, *Hesperosuchus agilis* [69], *Sphenosuchus acutus* [70]) is absent in *Riojasuchus tenuisceps* (PVL 3827, 3828), as is also the case in basal archosauriforms. *Riojasuchus tenuisceps* (PVL 3827, 3828) lacks a foramen on the medial side of the articular, contrasting with the condition in paracrocodylomorphs, aetosaurs, and *Euparkeria capensis* [36]. This probably results from the absence of the ventromedial process on the articular, which is the structure through which that foramen passes in archosaurs.

#### Cranial endocast

The endocranial cavity of *Riojasuchus tenuisceps* was studied based on the holotype specimen only because the referred specimen has its skull roof completely collapsed and is thus impossible to study that area by direct observation or CT scan images. The endocranial cavity of PVL 3827 is filled with a red, fine grained sandstone matrix that was not removed during its preparation, and therefore, the endocast of *Riojasuchus tenuisceps* was studied using a digital 3D model developed utilizing a CT scan of the skull (Fig 7).

In this digital endocast of *Riojasuchus tenuisceps*, the cerebral hemispheres are laterally expanded (Fig 7:Ceh), they are slightly longer than wide as in the aetosaurs *Neoaetosauroides engaeus* (PVL 5698; PULR 108) and *Aetosaurus ferratus* (SMNS 5775). On the other hand, the condition of *Riojasuchus tenuisceps* is different from that of the aetosaur *Desmatosuchus spurensis* [99] and the theropod *Sinraptor dongi* [19] whose cerebrum is approximately equally long as wide, or that of the extant *Alligator mississippiensis* (OUVC 9761) and the sauropod *Amargasaurus cazuai* [20] which is 1.2 to 2 times wider than long, respectively.

The width of the anterior brain of *Riojasuchus tenuisceps* almost triples from its anterior edge to the maximum width of the cerebral hemispheres, widening abruptly on the middle region of the hemispheres. This condition resembles that of *Alligator mississippiensis* (OUVC 9761), but contrasts with that of the aetosaurs *Neoaetosauroides engaeus* (PULR 108), in which the maximum width of the anterior brain only doubles from its anterior width, and *Desmatosuchus spurensis* [99], whose telencephalon slightly narrows posteriorly. The angle between the anterior and midbrain is approximately 130° (Fig 7), which does not differ much from that seen in the aetosaurs *Neoaetosauroides engaeus* (PULR 108) and *Desmatosuchus spurensis* [99] (ranging from 125° to 140°), the crocodile *Alligator mississippiensis* (140°) [100], the theropod dinosaur *Sinraptor dongi* [19], or the sauropod dinosaur *Amargasaurus cazaui* (140°) [20]. On the endocast of *Riojasuchus tenuisceps*, we identified the exit for the olfactory nerves (cranial nerves I) on the anterior end of the anterior brain, the trigeminal nerve (CN V) exiting laterally through a single foramen, the hypophysis on the ventral region of the anterior brain, and the base of the internal carotids arteries exiting from the ventral edge of the hypophysis (Fig 7;Olf, CN V, Hy, Inc). CN I seems to be short compared to that seen in theropod dinosaurs [101], but not as short as that of sauropod dinosaur [101]. The exit of the CN V through a single foramen suggests that the ophtalmic (V_1_), maxilar (V_2_), and mandibular (V_3_) branches of this nerve diverge once outside the braincase, resembling that of aetosaurs, many modern crocodiles and sauropod dinosaurs. It differs from that of some theropod dinosarus in which the branch V_1_ exits through a different foramen than V_2,3_ [101] and some fossil crocodiles (e.i. *Mourasuchus nativus*) in which the branch V_2_ exits through a different foramen than the V_3_ [102].

## Discussion and Conclusion

Ornithosuchids have been an enigmatic group of Triassic archosaurs with bizarre anatomical features and poorly known diversity. Their phylogenetic relationships have been quite controversial and debated for decades, but most recent studies regard them as basal pseudosuchians [1, 2, 4, 15, 103]. A better understanding of the anatomy of *Riojasuchus tenuisceps*, one of the most completely preserved species of this clade, will shed light on the interrelationships of Ornithosuchidae as well as their phylogenetic relationship within Archosauria.

In this contribution we reexamined the skull of the holotype and referred material of *Riojasuchus tenuisceps* and presented a detailed redescription of its cranial anatomy, which was previously studied by Bonaparte over 40 years ago [6, 17]. The skull of *Riojasuchus tenuisceps* has a very distinctive combination of features that distinguishes it from other archosaurs such as a diastema between the premaxilla and maxilla, the absence of a nasal-prefrontal contact, a jugal with a Y-shaped ascending process separating the antorbital fenestra from the infratemporal fenestra, the jugal participating in the antorbital fossa; the presence of palatine-pterygoid fenestrae, and a small tooth anterior to the two hypertrofied teeth on the dentary, among others. We recognized two synovial joints (basal and otic) but found no evidence of attachment of protractor musculature (e.g. preotic pendant) on the skull of *Riojasuchus tenuisceps* which, following the criteria of Holliday and Witmer [94], allowed us to infer a partially kinetically competence of the skull. This condition resembles that of non-avian saurischian dinosaurs, but differs from the kinetic avian dinosaurs and the akinetic modern crocodylians [94]. Trotteyn et al. [104] proposed that the pseudosuchians *Neoaetosauroides engaeus* and *Saurosuchus galilei* also have partially kinetic competent skulls, which would support the condition here interpreted for *Riojasuchus tenuisceps*. We also identified at least two autapomorphies on the skull of *Riojasuchus tenuisceps*, consisting of a suborbital fenestra equal in size to the palatine-pterygoid fenestra and a deep antorbital fossa that reaches the ventral margin of the maxilla.

Moreover, we made the first description of a digital endocast of *Riojasuchus tenuisceps*, which, although brief, will provide useful information for future studies on the subject contributing to the poor knowledge of palaeoneuroanatomy of pseudosuchians. The general shape of the encephalon of *Riojasuchus tenuisceps* does not differ much from that of the known pseudosuchians and theropods but it is more elongated than that of sauropod dinosaurs. The distribution of the recognized cranial nerves is consistent with that seen in other pseudosuchians, differing from theropod dinosaurs in some structures as the extension of the CN I and the branching of the CN V.

The revision of the phylogenetic affinities of *Riojasuchus tenuisceps* will be carried out in an upcoming contribution considering its postcranial anatomy, as well as the cranial features here described.
